# A spin torque meter with magnetic facet domains

**DOI:** 10.1038/s41467-018-06223-z

**Published:** 2018-09-17

**Authors:** Kyoung-Woong Moon, Changsoo Kim, Jungbum Yoon, Jun Woo Choi, Dong-Ok Kim, Kyung Mee Song, Dongseuk Kim, Byong Sun Chun, Chanyong Hwang

**Affiliations:** 1 Spin Convergence Research Team, Korea Research Institute of Standards and Science, Daejeon, 34113 Republic of Korea; 2 Center for Spintronics, Korea Institute of Science and Technology, Seoul, 02792 Republic of Korea; 3 Department of Physics, Soongsil University, Seoul, 06978 Republic of Korea; 4 Department of Physics, Sookmyung Women’s University, Seoul, 04130 Republic of Korea

## Abstract

Current-induced magnetic domain wall (DW) motion is an important operating principle of spintronic devices. Injected current generates spin torques (STs) on the DWs in two ways. One is the spin transfer from magnetic domains to the walls by the current flowing in the magnet. Current flow in attached heavy metals also generates another ST because of the spin-Hall effect. Both phenomena explain the wall motions well; therefore, their respective contribution is an important issue. Here, we show the simultaneous measurement of both torques by using magnetic facet domains that form mountain-shaped domains with straight walls. When the STs and the external magnetic field push the walls in opposite directions, the walls should have equilibrium angles to create balanced states. Such angles can be modulated by an additional in-plane magnetic field. Angle measurements distinguish the STs because each torque has a distinct mechanism related to the DW structure.

## Abstract (web summary)

Spin torques with different origins enable the magnetic domain wall motion, but the contributions are hard to be distinguished. Here, the authors show the method to measure both spin-magnetization transfer torque and spin–orbit torque by using mountain-shaped domains with straight domain walls.

## Introduction

The manipulation of the magnetization states by electric current injection is a significant issue in modern magnetism^1^ because it enables the application of data storage devices^1–4^, and provides numerous research topics^1,5–12^. The injected current generates the spin torque (ST) resulting from a conversion of charge current into spin current, which exerts a torque on the local magnetization. Measurements of ST are essential for the development of spintronic devices.

There are two types of STs (see Supplementary Note 1). One originates from transferring the spin angular momentum meditated by the current in the magnet^13–20^. The electric current flowing in the magnet has a polarized spin direction caused by the magnetization. When the polarized current flow feels a change in magnetization, the polarized current exerts a torque on the magnetization. Such an ST effect has been observed in a magnetic tunnel junction^13–15,20^, and on magnetic domain walls (DWs)^6,19,21,22^. In this paper, we will refer to this ST effect from magnetization changes as spin-magnetization-transfer torque (SMT). SMT is often also referred to as spin-transfer torque (STT)^3–5,13,14,17,18^, when the other STs are not considered. The other well-known ST is the spin–orbit torque (SOT)^23–29^ generated by ferromagnet/heavy metal interfaces. The electric current in the heavy metal produces a spin accumulation on the lateral surfaces of the heavy metal because of the spin-Hall effect^30–32^, and then the accumulated spins are pumped into the ferromagnet that generates the ST on the magnetization. This SOT effect has also been experimentally observed by DW motions^21,27–29,33,34^ and magnetization tiltings^25,26,35,36^. The origins of the two STs (SMT and SOT) are quite different, but estimation of the magnitude of the individual SMT and SOT is not simple because multilayered structures of the sample are needed to optimize the material parameters.

To measure the SMT and the SOT, several experimental methods are available. One of the most representative methods is the alternate current (AC) harmonic technique^35–37^, which detects the magnetization tilting of a magnetic domain induced by the SOT effect generated by an in-plane current flowing in heavy metal layers. Another measurement method, ST-ferromagnetic resonance (FMR)^38,39^, also detects the SOT. An in-plane AC current induces a steady precession of magnetization that results in a constant voltage drop caused by a phase difference between the current and the magnetoresistance. The ST-FMR method can also measure the SMT effect, but it requires a different sample geometry and a perpendicular current flow^15,40^. All of the above methods measure only one of either SMT or SOT in given sample structures.

**Fig. 1:**
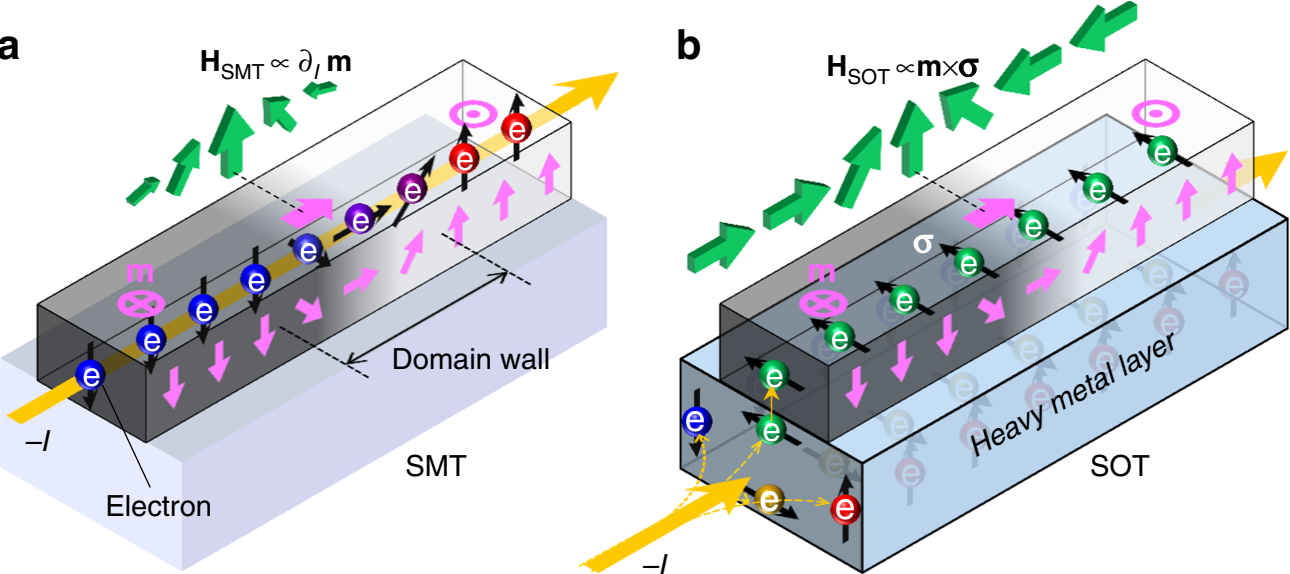
Two types of spin torque on magnetic domain walls. **a** An electric current *I* in the magnet feels the magnetization (**m**, pink arrows) variation at the DW that generates the SMT-induced magnetic field (**H**_SMT_). Here, **m** is a normalized vector. **b** The current in the attached heavy metal layer generates spin pumping into the magnet that produces the SOT-induced magnetic field (**H**_SOT_). **σ** is pumped spin direction (for more information on **H**_SMT_ and **H**_SOT_, see Supplementary Note 1)

The use of the DW enables the simultaneous measurement of SMT and SOT, because the DW has a magnetization change in itself^2,3,5–11^. The in-plane current in the magnetic layer senses the magnetization change that generates the SMT effect on the DW (Fig. 1a). In addition, the in-plane current flow in the heavy metal layer produces the SOT effect (Fig. 1b). Thus, if we measure the DW motion, we can obtain the total ST ( = SMT + SOT) effect. In this case, however, separation of SMT and SOT is laborious^21^. Note that, most of these experiments are performed on patterned wire structures to simplify the DW position.

In this study, we show a method for quantifying the total ST as well as the separation between SMT and SOT by observing the magnetic domain shapes in two-dimensional films (not in wires). Application of certain magnetic fields and current produce unusual magnetic domains that have clear and straight DWs. That is a facet domain^41,42^. In this paper, we will focus on the nonadiabatic SMT and the damping-like SOT because they produce effective perpendicular magnetic fields for wall motion in our system (see Supplementary Note 2).

## Results

### Facet domain formation

**Fig. 2:**
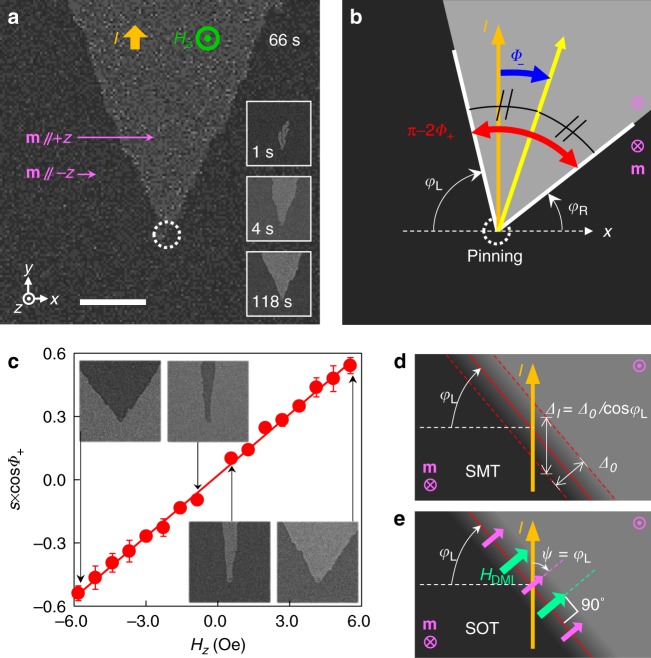
Facet domain formation. **a** Facet domain formation after nucleation at nucleation sites (white dashed circles). Current *I* = +0.15 A and the perpendicular field *H*_*z*_ = +4.1 Oe. Dark (light) gray represents –*z* (+*z*) magnetization. Insets show the magnetization states at different time. The scale bar is 10 µm. **b** Definition of two magnetization angle (*φ*_R_ and *φ*_L_) of DWs. The yellow arrow shows the center direction of the facet domain. The red and blue arrows show alternative angle definitions for *Φ*_+_ and *Φ*_–_. **c** Facet angles with respect to *H*_*z*_ under a fixed current (*I* = +0.15 A). Error bars were obtained from more than five measurements. The red line is a linear fit and the possible error in the linear fit produces the uncertainty of *H*_ST_. Insets show facet images at different values of *H*_*z*_. **d** Mechanism of the SMT-only facet. The pink arrow indicates the magnetization direction. Red dashed lines show the DW width (*Δ*_0_). **e** Mechanism of the SOT-only facet. The DMI field (*H*_DMI_, green arrow) aligns the magnetization of DW (pink arrow at DW)

The formation of a magnetic facet domain over time upon the application of an electric current (*I*) and an external perpendicular magnetic field (*H*_*z*_) is shown in Fig. 2a. The magnetization states of the sample were observed by a magneto-optical Kerr effect microscope^42,43^. The sample has ultrathin heterostructures (see Methods) and exhibits a perpendicular magnetic anisotropy (PMA), and thus, only the +*z* (–*z*) magnetization domain is shown as light (dark) gray. The facet domain formed as follows. First, we turned on *I* flowing in the +*y* direction and applied *H*_*z*_ to fully saturate the magnetization in the –*z* direction. Next, we changed the direction of *H*_*z*_ to the +*z* direction at time zero, then the domains in the +*z* direction nucleate at nucleation sites (white circles) and grow in the current direction (insets of Fig. 2a). The growth of +*z* domains formed hilly domains that maintain their shape. We call this a facet domain^41,42^ because it has straight and clear edges.

Because of the clear edges, we can obtain the DW angle (*φ*) on the right (*φ*_R_) and left (*φ*_L_) sides of the nucleation site (Fig. 2b). There is no guarantee that these two angles will be the same, it is useful to introduce alternative angle definitions, such as *Φ*_+_ = (*φ*_R_ + *φ*_L_)/2 and *Φ*_−_ = (*φ*_R_ − *φ*_L_)/2. Here, *Φ*_+_ (red arrow, shows π − 2*Φ*_+_) indicates the sharpness of the facet domains and *Φ*_−_ (blue arrow) represents the tilting of the center line (yellow arrow) of the facet domains from the current direction.

We measured the facet domains as a function of *H*_*z*_ with fixed *I*. The inset of Fig. 2c shows several facet domains showing a clear change in *Φ*_+_ depending on *H*_*z*_, but *Φ*_−_ is almost constant (~0). So, we only obtained *s* × cos*Φ*_+_ as a function of *H*_*z*_, where *s* is the domain polarity along the direction of *I* (+*y*). When *I* passes through the DW from a ∓*z* domain to a ±*z* domain, *s* is ±1. The measured *s* × cos*Φ*_+_ is shown in Fig. 2c as a function of *H*_*z*_, exhibiting a linear correlation.

This linear correlation can be explained by the SMT effect based on an effective DW width. The magnetization rotates from ±*z* to ∓*z* within a short length scale (~10 nm) between two domains in the PMA films. This scale is known as the DW width (*Δ*_0_) and is determined by material parameters. The DW width is a crucial parameter of the SMT because the strength of the SMT-induced field is proportional to the magnetization gradient along the current flowing in the magnetic layer^16–18^ (Fig. 1a). The gradient is inversely proportional to the DW width. When the DW is tilted from the current, the current feels the effective DW width (*Δ*_*I*_), which has a cos *φ* dependence (Fig. 2d). A similar description is given by Moon et al.^42^ by using the concept of a current component normal to the DW.

**Fig. 3:**
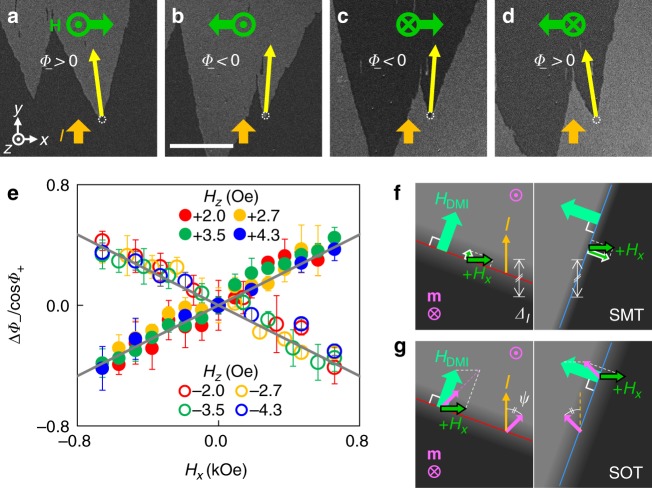
Tilting of the facet by additional *H*_*x*_. **a**–**d** Tilted facets with different field directions under a fixed current (*I* = +0.15 A). **H** = (*H*_*x*_,*H*_*y*_,*H*_*z*_) is the external magnetic field. |*H*_*x*_| = 0.56 kOe, |*H*_*y*_| = 0, and |*H*_*z*_| = 3.5 Oe. Field directions are shown in the images as green arrows. Yellow arrows represent center direction of the facet domains. The scale bar is 50 µm. **e** Facet tilting versus *H*_*x*_ under a fixed current (*I* = +0.15 A). |*H*_*z*_| are 2.0 (red circles), 2.7 (orange circles), 3.5 (green circles), 4.3 Oe (blue circles). The sign of *H*_*z*_ is positive (negative) for closed (open) circles. The gray lines are linear fits. Error bars were obtained from more than five measurements. **f** Tilting mechanism of the SMT-only facet at left (left panel) and right (right panel) sides of the facet. The white-lined green arrows are the parallel component of *H*_*x*_ to *H*_DMI_. **g** Tilting mechanism of the SOT-only facet at left (left panel) and right (right panel) sides of the facet. The sum of *H*_DMI_ and *H*_*x*_ is aligned in the direction of DW magnetization (pink arrow at DW)

Besides the SMT, the SOT also can describe the facet domain, but the latter has a distinct mechanism. The SOT-induced effective field is determined by the parallel component of the magnetization along the direction of current flowing in the upper or under layers (Fig. 1b), but not in the magnetic layer^27–29,33,34^. The DW should have a pure in-plane magnetization at the center of DW with an angle (*ψ* in Fig. 2e). We define *ψ* as the angle between the magnetization and the direction of *I*. Thus, the SOT-induced field is proportional to cos*ψ*. Generally, the DW magnetization angle (*ψ*) and the DW angle (*φ*) need not be related, however the interfacial Dzyaloshinskii–Moriya interaction (DMI)^44,45^ produces an effective in-plane field known as the DMI field^28,46^ (*H*_DMI_), which is normal to the DW (Fig. 2e). Thus, a sufficiently large *H*_DMI_ can align *ψ* to *φ* to explain the linear behavior shown in Fig. 2e.

### Total ST

From the linear dependence of the SMT and SOT on cos*φ*, we can obtain the total ST-induced field as:

1 $$s \times \cos {\it{\Phi }}_ + = - \frac{{H_z}}{{H_{{\mathrm{ST}}}}} \cdot$$

Here, *H*_ST_ is the total ST-induced field when *φ* = *ψ* = 0 (normal incidence of *I* on the DW). The obtained value of *H*_ST_ was –10.3 ± 0.2 Oe at *I* = + 150 mA. A negative value of *H*_ST_ means that the current from the ∓*z* domain to the ±*z* domain generates a field in the ∓*z*-direction that pushes the DW in the current direction. We know that *H*_ST_ is composed of the SMT-induced field (*H*_SMT_) and the SOT-induced field (*H*_SOT_) (*H*_ST_ = *H*_SMT_ + *H*_SOT_). However, at this stage, we have no information on the *H*_SMT_ and *H*_SOT_ parts. To separate these two effects, we applied an additional in-plane magnetic field during the facet measurements (for more details on facet formation see Supplementary Note 2).

### Facet tilting

The facet domains under an additional in-plane magnetic field to the *x*-direction are shown in Fig. 3a–d. The addition of an in-plane field in the *x*-direction (*H*_*x*_) tilts the facet domains from the current direction, i.e., *Φ*_−_ is a function of *H*_*x*_; *Φ*_−_(*H*_*x*_). In contrast to *Φ*_−_, *Φ*_+_ does not show meaningful variations. We define this phenomenon as a facet tilting. Figure 3e shows the change in *Φ*_−_(*H*_*x*_) with several *H*_*z*_ and fixed *I* = +0.15 A. Here, Δ*Φ*_*−*_ = *Φ*_*−*_(*H*_*x*_)–*Φ*_*−*_(*H*_*x*_ = 0). Interestingly, if we normalize Δ*Φ*_*−*_ by cos*Φ*_*+*_, such as Δ*Φ*_*−*_/cos*Φ*_*+*_, all the results exhibit a single linear correlation with *H*_*x*_. Such a correlation only changes sign depending on the domain polarity

To describe the facet tilting based on the SMT, we should consider the variation in the width of the DW. The applied in-plane magnetic field, which is parallel to *H*_DMI_, changes the DW width such as *Δ*_0_[1 + (π*H*_ǁ_)/(2*H*_*K*_)+…], because *H*_ǁ_ prefers an expanded DW (see ref.^47^ or Supplementary Note 3). Here, *H*_ǁ_ is the component of *H*_*x*_ parallel to *H*_DMI_ and *H*_*K*_ is the effective anisotropy field of PMA. It is known that *H*_DMI_ has a chirality that prefers a certain normal direction to the DW^28,44–46^. Thus, the applied *H*_*x*_ produces opposite *H*_ǁ_ components on each side of the facet that makes a different wall width on each side (Fig. 3f). Regardless of the different wall widths, the current should feel the same gradient of the magnetization, in other words, the same *Δ*_*I*_. Therefore, *φ*_R_ and *φ*_L_ should have different values. After some calculation, we obtained the facet-tilting equation for the SMT-only case as *s* × Δ*Φ*_−_/cos*Φ*_+_ ≅ (π*H*_*x*_)/(2*H*_*K*_). Recent studies show that *H*_SMT_ depends on *Δ*_0_ but the variation is much larger than that of *Δ*_0_. The wall width variation should induce an additional change of SMT because there is a possibility that the nonadiabatic coefficient of SMT depends on the wall width^21^. So, if we observe a significant difference in *H*_*K*_ values measured by the facet method or by other methods, this would be another evidence for their expectation. To include this possibility, we replaced *H*_*K*_ with ${H_K}^\ast$ (for more details on the facet tilting see Supplementary Note 3).

The SOT-only case can also produce facet tilting. To compensate for the external *H*_*z*_ on the DW, the SOT-induced field should have the same value regardless of the left or right side of the facet. This means that *ψ* should have a constant value. Figure 3g shows how *H*_*x*_ and *H*_DMI_ form a stable state. The summation of two vectors, *H*_*x*_ and *H*_DMI_, should align in the DW magnetization direction that requires different values of *φ*_R_ and *φ*_L_. From this assumption, we obtain the facet-tilting equation of the SOT-only case as *s* × Δ*Φ*_−_/cos*Φ*_+_ ≅ *H*_*x*_/*H*_DMI_ (for more details on the facet tilting see Supplementary Note 3).

Combining the above two equations on facet tilting, we obtain the facet-tilting equation by ST. Assuming that the variations in all angles are small, the facet-tilting effect of SMT and SOT should be determined by the proportion of each effect in ST, and then the equation is:

2 $$s \times \frac{{\Delta {\it{\Phi }}_ - }}{{\cos {\it{\Phi }}_ + }} \cong \left( {\frac{{H_{{\mathrm{SOT}}}}}{{H_{{\mathrm{ST}}}}}\frac{1}{{H_{{\mathrm{DMI}}}}} + \frac{{H_{{\mathrm{SMT}}}}}{{H_{{\mathrm{ST}}}}}\frac{{\mathrm{\pi }}}{{2{H_K}^\ast }}} \right)H_x \cdot$$

If we know any two values from among *H*_SOT_, *H*_SMT_, *H*_DMI_ and ${H_K}^\ast$, we can separate *H*_SOT_ and *H*_SMT_ from Eqs. (1) and (2). Independent measurements obtain *H*_SOT_ = –15.4 ± 0.9 Oe and *H*_DMI_ = 1.25 ± 0.12 kOe (see Supplementary Note 5). As a result, we obtained *H*_SMT_ = + 5.1 ± 1.1 Oe and ${H_K}^\ast$ = 1.3 ± 0.8 kOe at *I* = + 0.15 A. These values mean that the SOT effect pushes the DW in the current direction but the SMT effect pushes the DW in the opposite direction in our sample. We note that ${H_K}^\ast$ is ~4.8 times smaller than *H*_*K*_ (6.1 ± 0.3 kOe) measured by other method (see Supplementary Note 6), and this is another evidence for the dependence of the nonadiabatic coefficient of SMT (*β*) on the DW width^21^.

### Facet sharpening

**Fig. 4:**
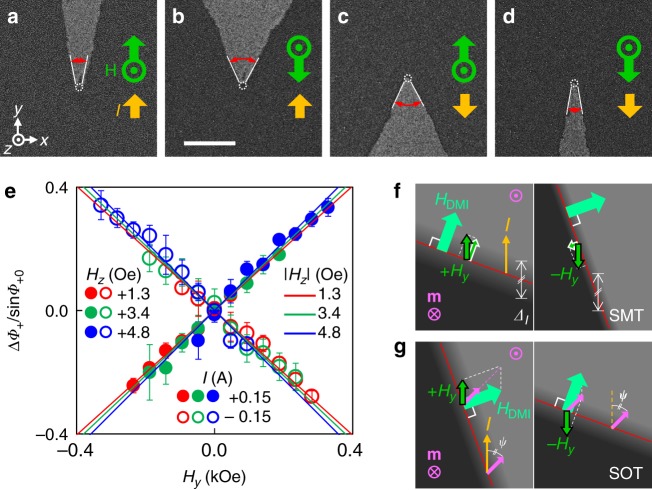
Sharpening of the facet by additional *H*_*y*_. **a**–**d** Facets with different fields under a fixed current (|*I|* = 0.15 A). |*H*_*x*_| = 0, |*H*_*y*_| = 0.14 kOe, and |*H*_*z*_| = 3.4 Oe. Field and current directions are shown in the images. The scale bar is 20 µm. **e**, Facet sharpening as a function of *H*_*y*_. *H*_*z*_ are 1.3 (red circles), 3.4 (green circles), and 4.8 Oe (blue circles). *I* are +0.15 A for closed circles and −0.15 A for open circles. Error bars were obtained from more than five measurements. **f** Sharpening mechanism of the SMT-only facet at a left side of the facet under +*H*_*y*_ (left panel) and –*H*_*y*_ (right panel). **g** Sharpening mechanism of the SOT-only facet at a left side of the facet under +*H*_*y*_ (left panel) and –*H*_*y*_ (right panel)

To confirm these ST effects, we performed other experiments with the in-plane field in the *y*-direction (*H*_*y*_) under *I* and several values of *H*_*z*_. *H*_*y*_ mainly changes *Φ*_+_ and no significant facet tilting is observed (Fig. 4a–d). We define this phenomenon as a facet sharpening. These sharpening experiments should be a complementary method for measuring the ST effects thus these experiments can countercheck the results of facet tilting. We measured *Φ*_+_ with respect to *H*_*y*_; *Φ*_+_(*H*_*y*_). The facet sharpening is normalized as *s* × Δ*Φ*_+_/sin*Φ*_+0_ and the values correlate almost linearly with *H*_*y*_ (Fig. 4e). Here, Δ*Φ*_+_ = *Φ*_+_(*H*_*y*_)–*Φ*_+_(*H*_*y*_ = 0) and *Φ*_+0_ = *Φ*_+_(*H*_*y*_ = 0).

The effect of SMT on the facet sharpening with *H*_*y*_ is shown in Fig. 4f. +*H*_*y*_ has a component parallel to *H*_DMI_, thus the DW has an elongated width but −*H*_*y*_ reduces the DW width because of the antiparallel component to *H*_DMI_. It is clear that these dependences do not distinguish between the left and right sides of the facet, thus only the variations in *Φ*_+_ have a meaning. After some calculation and simplification, we obtain *s* × Δ*Φ*_+_/sin*Φ*_+0_ ≅ −(π*H*_*y*_)/(2*H*_*K*_ tan^2^*Φ*_+0_), which is the facet-sharpening equation for the SMT-only case. Note that the right-hand side of the equation is negative.

The SOT-only case is shown in Fig. 4g. The vector summation of *H*_DMI_ and *H*_*y*_ should result in a constant *ψ* that requires a different *φ* (or *Φ*_+_) with respect to *H*_*y*_. From this description, we derived the facet-sharpening equation for the SOT-only case as *s* × Δ*Φ*_+_/sin*Φ*_+0_ ≅ *H*_*y*_/*H*_DMI_. The right-hand side of the equation is positive and this differs from the SMT-sharpening equation.

From these two facet-sharpening equations, the ST-induced facet sharpening equation is obtained as follows:

3 $$s \times \frac{{\Delta {\it{\Phi }}_ + }}{{\sin {\it{\Phi }}_{ + 0}}} \cong \left[ \begin{array}{l}\frac{{H_{{\mathrm{SOT}}}}}{{H_{{\mathrm{ST}}}}}\frac{1}{{H_{{\mathrm{DMI}}}}}\left( {1 - \frac{{C_2}}{{\sin ^2{\it{\Phi }}_{ + 0}}}\frac{{|H_{{\mathrm{DMI}}}|}}{{H_K}}} \right)\\ - \frac{{H_{{\mathrm{SMT}}}}}{{H_{{\mathrm{ST}}}}}\frac{{\mathrm{\pi }}}{{2{H_K}^ \ast \tan ^2{\it{\Phi }}_{ + 0}}}\end{array} \right]H_y \cdot$$

Here, *C*_2_ is a correction factor come from the domain tilting (see Supplementary Note 4). Micromagnetic simulations^48^ show 0.8 is the best value for *C*_2_ (see next section and Supplementary Note 7). This equation has an angular dependence on *Φ*_+0_ in the right side. Linear lines with red, green, and blue colors in Fig. 4e represent the expected values for each *Φ*_+0_. Within our uncertainty range the expected values correspond well to the sharpening results. Thus, our experiments show quite consistent results.

### Micromagnetic simulation

**Fig. 5:**
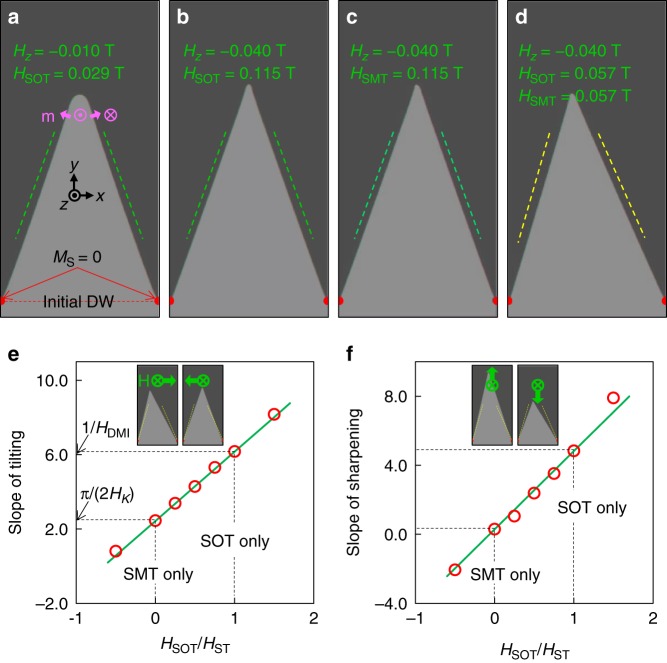
Micromagnetic simulations. **a**–**d** Dark (light) gray represents –*z* (+*z*) magnetization. Red areas are strong defects (*M*_S_ = 0). Green dashed lines represent the angle cos^–1^(*H*_*z*_/*H*_ST_). Yellow dashed lines show the angle rotated 4° from the green dashed lines. **e** Slopes of tilting. **f** Slopes of sharpening. Red circles are the data. Black dashed lines depict SOT-only and SMT-only cases. Green lines are linear interpolations between SOT-only and SMT-only cases. Insets show examples of the facet with *H*_SOT_/*H*_ST_ = 0.5, *H*_*z*_ = −0.04 T, and |*H*_*x*_| = |*H*_*y*_| = 0.04 T. Green arrows depict the direction of magnetic field

These experimental results are reproduced by micromagnetic simulations^48^ (see Methods). The initial magnetizations are a straight DW (dashed red line in Fig. 5a) connecting two defects (half red circles in Fig. 5a–d). If we change *H*_*z*_ and *H*_ST_, the domains converge to the facet domains and the converged *Φ*_+_ depend only on the ratio of *H*_*z*_/*H*_ST_ (Fig. 5a–c). Small value of *H*_*z*_ and *H*_ST_ make a blunt vertex because the influence of the DW tension^49^ is relatively large at the facet vertex. If we apply *H*_SOT_ and *H*_SMT_ simultaneously, we can see an offset of tilting because the adiabatic SMT tilts the DW magnetization that changes the SOT field at each side of the facet (Fig. 5d).

Next, the simulations confirm the universality of the facet tilting and sharpening with different parameter sets (see Supplementary Note 7). The tilting of SOT-only facets shows a linear dependence of sin*Φ*_−_/cos*Φ*_+_ on *H*_*x*_, as expected by Supplementary Equation (10) (Supplementary Note 3). The sharpening results also show a clear linear relation between sin(*Φ*_+_ – *Φ*_+0_)/sin*Φ*_+0_ and *H*_*y*_, as described by Supplementary Equation (17) (Supplementary Note 4). From the sharpening simulation, we obtained *C*_2_ = 0.8 for Eq. (3). SMT-only facets also show similar behavior to the SOT (see Supplementary Note 7). Tilting of facets shows a good linear behavior of sin*Φ*_−_/cos*Φ*_+_ on *H*_*x*_, as expected by Supplementary Equation (6) (Supplementary Note 3), but the results of sharpening show a significant quadratic dependence of sin(*Φ*_+_ − *Φ*_+0_)/sin*Φ*_+0_ on *H*_*y*_. Despite these quadratic dependences, the curves have a tangent line with a slope near *H*_*y*_ = 0, as expected by Supplementary Equation (13) (Supplementary Note 4). It is therefore useful for obtaining the linear relation of sharpening that derives an odd function such as *f*_odd_ (+*H*_*y*_) = {*f*(+*H*_*y*_) − *f*(−*H*_*y*_)}/2.

Finally, we test the linear interpolation of the facet tilting and sharpening between SOT-only and SMT-only facets. For these tests, we fixed *H*_ST_ = (*H*_SOT_ + *H*_SMT_) = 0.115 T and *H*_*z*_ = −0.04 T. We changed the relative portion of *H*_SOT_ in *H*_ST_ so that *H*_SMT_ =  *H*_ST_ − *H*_SOT_. Figure 5e and f shows the slope of tilting (rate of change of sin *Φ*_−_/cos*Φ*_+_ as a function of *H*_*x*_) and the slope of sharpening (rate of change of sin (*Φ*_+_ − *Φ*_+0_)/sin*Φ*_+0_ as a function of *H*_*y*_). The results show that the linear interpolation is good for estimating the tilting and sharpening of facets. In detail, the tilting of facets exhibits almost perfect linear interpolation but the sharpening show small deviation from the linear interpolation. So it is better for using facet tilting rather than sharpening to determine the spin-torque parameters. We think the main cause is that the tilting is obtained by the difference of wall angles that naturally subtracts the quadratic dependence on applied field. The sharpening experiments should be used as an auxiliary method.

## Discussion

We perform the facet experiments on other samples having a different thickness of Pt layer inserted between CoFeB and MgO layers. (see Supplementary Notes 1,0 and 1,1). The other samples also show the clear facet domains, though the samples prefer stripe domain states. Table 1 shows summarized STs and related fields. Thicker Pt layer insertion reduces the total ST effect. We think that the SOT effect of the lower Pt and the upper Pt layers cancel each other out. The SMT effect also decreases with thicker inserted Pt layer because reduced *H*_*K*_ increase the wall width.

**Table 1:** ST effects with Pt/CoFeB/Pt(*x* nm)/MgO stacks

	*x* = 0.0 nm	*x* = 0.4 nm	*x* = 0.6 nm
*ε*_ST_ (10^−14^ T m^2^ A^−1^)	−11.8 ± 1.0	−6.4 ± 0.1	−4.0 ± 0.1
*ε*_SOT_ (10^−14^ T m^2^ A^−1^)	−15.4 ± 2.9^b^	−9.6 ± 0.6	−5.7 ± 0.3
*ε*_SMT_ (10^−14^ T m^2^ A^−1^)	3.6 ± 1.9^a^	3.2 ± 0.7	1.7 ± 0.4
*H*_DMI_ (kOe)	3.1 ± 0.8^b^	1.25 ± 0.12	0.47 ± 0.03
*H*_*K*_ (kOe)	6.4 ± 0.2	6.1 ± 0.3	2.2 ± 0.1
*H*_K_^*^ (kOe)	0.5 ± 0.3^b^	1.3 ± 0.8	0.24 ± 0.08
Temp. (K)	~305	~320	~320

This table shows the ST efficiencies, related fields, and temperatures. The samples are Sub/Ta(3 nm)/Pt(3 nm)/Co_40_Fe_40_B_20_(0.9 nm)/Pt(*x* = 0.0 nm)/MgO(1.5 nm)/Ta(2 nm), Sub/Ta(3 nm)/Pt(3 nm)/Co_40_Fe_40_B_20_(0.9 nm)/Pt(*x* = 0.4 nm)/MgO(1.5 nm)/Ta(2 nm), and Sub/Ta(3 nm)/Pt(3 nm)/Co_40_Fe_40_B_20_ (0.9 nm)/Pt(*x* = 0.6 nm)/MgO(1.5 nm)/Ta(2 nm)/Pt (1.5 nm). *ε*_ST_( = *H*_ST_/*J*), *ε*_SOT_( = *H*_SOT_/*J*), and *ε*_SMT_( = *H*_SMT_/*J*) are the spin torque efficiencies, where *J* is the current density. We assume a uniform current density in all layers except for the MgO layer.

^a^*ε*_SMT_ is an interpolated value from the values of *x* = 0.4 nm sample (see Supplementary Note 1).

^b^Expectation values obtained from *ε*_SMT_, *ε*_ST_, and *H*_*K*_

Our scope is valid until the DMI is strong enough for keeping the Néel type DW configuration. If the DMI is weaker than the Néel type demagnetization field, the wall type is an intermediate DW between Bloch and Néel configurations^50^. Such configuration can have two magnetization angles of the DW which are energetically same. So, we think such situation is not good for micromagnetic simulations assuming ideal samples because the simulation should only show one angle of the wall magnetization or strange steady states continuously changing between two stable angles of the wall. If we want to reproduce this weak DMI case by simulations, we have to consider pinning distributions for independent segment length of DW and relative stability of each magnetization angle of wall under thermal activation. This requires many unknown assumptions, which makes the solution of this problem impossible. However, we suggest a crude model for future works (Supplementary Note 1,2).

Our discovery of the magnetic facet domain obtains the main parameters of spintronics. The magnetic facet domain is formed by competition between the external perpendicular field and the electric current that directly shows the total ST strength from stabilized facet angles. The application of an additional in-plane field during facet formation shows two different phenomena, namely facet tilting and facet sharpening. These two phenomena can be induced not only by SMT, but also by SOT. However, SMT and SOT have separate distinct origins. The DW width variation by the in-plane field is the core of the SMT-induced facet tilting and sharpening. In contrast, the magnetization angle of DW is the main cause of the SOT-induced facet tilting and sharpening. Through these distinct mechanisms, we can determine the individual strength of ST: SMT and SOT.

## Methods

### Sample and measurement

A multilayer stack consisting of Ta (3 nm)/Pt (3 nm)/Co_40_Fe_40_B_20_ (0.9 nm)/Pt (0.4 nm)/MgO (1.5 nm)/Ta (2 nm) was deposited on a Si/SiO_2_ substrate by a magnetron sputtering system. The base pressure of the sputtering chamber was 5 × 10^−^^9^ Torr. An ion-milling process produced wire structures of 1 mm width. Using photolithography, Ti (5 nm)/Au (100 nm) electric contact pads were deposited on the wires with 200 μm spacing. We passed the electric current through the contact pads and obtained an area (200 μm long and 1 mm wide) where the electric current had a uniform density. The total current was fixed at 0.15 A and the positive current direction was set to +*y* in this study. We performed all the experiments at room temperature, but Joule heating increased the sample temperature with the current passing. We estimated the sample temperature to be ~320 K measured by a thermocouple placed on the sample. We believe that such a temperature increase does not make a significant difference in the STs of room temperature (see Supplementary Notes 8 and 9). Magnetic field alignment is important in this experiment. The applied in-plane field (*H*_*x*_ and *H*_*y*_) was in the order of several hundred Oe, but the order of the perpendicular field (*H*_*z*_) was several Oe. Thus, misalignment of the in-plane field can generate a significant perpendicular field. To align the in-plane field, the sample was attached to a motorized tilting stage with a resolution angle of less than 0.001°. We could confirm the alignment from the facet sets shown in Figs. 3a–d and 4a–d. The facet sets cannot be explained by the field misalignment.

### Micromagnetic simulation

We performed micromagnetic simulations by using MUMAX3^48^. The simulation geometry is 2 μm × 4 μm × 1 nm with a 4 nm × 4 nm × 1 nm cell and a periodic boundary condition to the *x*-axis. The material parameters are as follows. The saturation magnetization (*M*_S_) is 900 × 10^3^ A m^−1^, the exchange stiffness constant (*A*) is 1 × 10^−11^ J m^−1^, the anisotropy constant (*K*) is 0.8 × 10^6^ J m^−3^, and the interfacial DMI (*D*) is –1 mJ m^−2^. The geometry has defects where the saturation magnetization is 0 for strong pinning of the DWs. We set the field-like SOT at 0. For fast stabilization, the damping constant (*α*) is adjusted from 0.1 to 10. We assume the current pushes the DW to the +*y* direction. We consider the nonadiabatic coefficient of SMT has no dependence on the wall width.

## Electronic supplementary material

Supplementary Information

## Data Availability

The data that support the plots in this paper and other findings of this study are available from the corresponding author upon reasonable request.
