# Supplementary material for: A spin torque meter with magnetic facet domains
Source: Nat Commun. 2018 Sep 17;9:3788. doi: 10.1038/s41467-018-06223-z (PMC6141574; doi:10.1038/s41467-018-06223-z)
Supplement: Supplementary file 1 — Supplementary Information [file 41467_2018_6223_MOESM1_ESM.pdf]

Supplementary Information for
Spin torque meter with magnetic facet domains

Kyoung-Woong Moon[1], Changsoo Kim[1], Jungbum Yoon[1], Jun Woo Choi[2], Dong-Ok Kim[2,3], Kyung Mee Song[2,4], Dongseuk Kim[1], Byong Sun Chun[1] & Chanyong Hwang[1,*]

[1]*Spin Convergence Research Team, Korea Research Institute of Standards and Science, Daejeon 34113, Republic of Korea*

[2]*Center for Spintronics, Korea Institute of Science and Technology, Seoul 02792, Republic of Korea*

[3]*Department of Physics, Soongsil University, Seoul 06978, Republic of Korea*

[4]*Department of Physics, Sookmyung Women's University, Seoul 04130, Republic of Korea*

*Email: cyhwang@kriss.re.kr

Table of contents

1. Supplementary Note 1: ST in LLG equation 2

2. Supplementary Note 2: Facet formation 3

3. Supplementary Note 3: Facet tilting 4

4. Supplementary Note 4: Facet sharpening 6

5. Supplementary Note 5: Estimation of H_{SOT} and H_{DMI} 8

6. Supplementary Note 6: Estimation of H_K 10

7. Supplementary Note 7: Micromagnetic simulations for SOT- and SMT only facet 11

8. Supplementary Note 8:Temperature and Oersted field problem 14

9. Supplementary Note 9: Compare with other method 15

10. Supplementary Note 10: Other sample 1 17

11. Supplementary Note 11: Other sample 2 21

12. Supplementary Note 12: Weak DMI case 24

Supplementary Note 1

: Spin torque (ST) in the Landau–Lifshitz–Gilbert (LLG) equation

Magnetization dynamics is described by the LLG equation including ST as follows[1-11].

$$\dot{\mathbf{m}} = \begin{bmatrix} -\gamma_0 \mathbf{m} \times \mathbf{H_{eff}} & +\alpha \mathbf{m} \times \dot{\mathbf{m}} \\ +\mathbf{m} \times [\mathbf{m} \times \{(\mathbf{u} \cdot \nabla)\mathbf{m}\}] & +\beta \mathbf{m} \times \{(\mathbf{u} \cdot \nabla)\mathbf{m}\} \\ +\gamma_0 \tau_{\mathrm{d}} \mathbf{m} \times (\mathbf{m} \times \boldsymbol{\sigma}) & +\gamma_0 \tau_{\mathrm{f}} \mathbf{m} \times \boldsymbol{\sigma} \end{bmatrix} \quad (1)$$

$$= \begin{bmatrix} \text{field torque} & \text{damping torque} \\ \text{adiabatic SMT} & \text{non} - \text{adiabatic SMT} \\ \text{damping} - \text{like SOT} & \text{field} - \text{like SOT} \end{bmatrix}.$$

The first line of the right side of the equation is the terms of a conventional LLG equation: field torque and damping torque. The second line represents two kinds of spin torque induced by magnetization variations (SMT)[1-4]: adiabatic SMT and non-adiabatic SMT. The last line shows two kinds of spin–orbit torque (SOT)[5-11]: damping-like SOT and field-like SOT. The symbols are: $\mathbf{m}$, the unit vector of local magnetization, $\dot{\mathbf{m}}$, the time derivative of $\mathbf{m}$, γ_0, the gyromagnetic constant, $\mathbf{H_{eff}}$, the effective magnetic field vector which includes external, exchange, anisotropy, DMI, and demagnetization, α, the damping constant, $\mathbf{u}$, the velocity vector of SMT, β, the non-adiabatic coefficient of SMT, τ_{d}, the coefficient of damping-like SOT, $\boldsymbol{\sigma}$, the unit vector of the spin direction pumped from other layers (except for the magnetic layer) due to the spin Hall effect, and τ_{f}, the coefficient of field-like SOT. Note that $\mathbf{u}$ is proportional to the current in the magnetic layer but, τ_{d} and τ_{f} are proportional to the current in the attached layer (not the magnetic layer). $\boldsymbol{\sigma}$ is transverse to the current direction.

Supplementary Note 2

: Facet formation

Supplementary Equation (1) is valid for magnetization dynamics of perfect samples (without pinning). Typical samples have many pinning sites due to sample irregularity that drastically reduces the speed of DW motions known as the DW creep[3,12]. However, non-zero field still produces non-zero speed in the creep regime[3,12]. Thus, all the driving force should be cancelled to stop DWs. Such conditions are easily obtained by inserting $\dot{\mathbf{m}} = 0$ in Supplementary Equation (1). This means that a cross product of total field and $\mathbf{m}$ should be zero as follows:

$$0 = -\gamma_0 \mathbf{m} \times \begin{bmatrix} \mathbf{H_{eff}} & 0 \\ -(1/\gamma_0)\mathbf{m} \times \{(\mathbf{u} \cdot \nabla)\mathbf{m}\} & -(1/\gamma_0)\beta(\mathbf{u} \cdot \nabla)\mathbf{m} \\ -\tau_{\mathrm{d}}(\mathbf{m} \times \boldsymbol{\sigma}) & -\tau_{\mathrm{f}}\boldsymbol{\sigma} \end{bmatrix}. \quad (2)$$

Applying the current (I) and perpendicular field (H_z) makes the facet shown in Fig. 2. The DW always has pure in-plane magnetization at the DW centre that produces nonzero torque due to H_z. To compensate this effect, ST should produce $-H_z$. The adiabatic SMT and the field-like SOT term do not produce perpendicular field, thus only the non-adiabatic SMT and the damping-like SOT cancel H_z. These two terms have clear angular dependences explained in the main manuscript. The DW tilting changes the magnetization gradient along the current direction. Tilted DW magnetization also changes the $\mathbf{m} \times \boldsymbol{\sigma}$ value because $\boldsymbol{\sigma}$ is transverse (+x or –x in this paper) to the current direction (+y). We can define the strength of SMT-induced perpendicular field ($|H_{\mathrm{SMT}}|$) as $\beta u/(\gamma_0 \Delta_0)$, where Δ_0 is the DW width. The strength of the SOT-induced perpendicular field ($|H_{\mathrm{SOT}}|$) is $(\pi/2)\tau_{\mathrm{d}}$. Here, $\pi/2$ is required due to integration over the DW. Note that, to determine the sign of H_{SMT} and H_{SOT}, the domain polarity (s) and the DMI field (H_{DMI})[12-14] direction are needed. However, for simplicity, we selected a situation shown in Figs 2–4. The current flows to the +y direction and feels the change of –z domain to +z domain. The directions of vectors are: H_z is +z, H_{SMT} and H_{SOT} are –z to cancel H_z, H_{DMI} points to +z domain from –z domain, and the DW magnetization is parallel to H_{DMI}.

Supplementary Note 3

: Facet tilting

The SMT-induced facet tilting requires DW width variation. Ref. 15 described the DW width as a function of parallel field ($H_{\parallel}$) to H_{DMI} such that $\Delta_0/\left(\sqrt{1-h^2}-h\cos^{-1}h\right)$, where h is $H_{\parallel}/H_K$ and H_K is an effective anisotropy field of PMA. Using the Taylor expansion, we obtain $\Delta_0\left[1+(\pi H_{\parallel})/(2H_K)+(\pi^2-2){H_{\parallel}}^2/(2H_K)^2+\cdots\right]$. The SMT-only facet has a stabilized angle without the in-plane field:

$$H_{\mathrm{SMT}}\cos\varphi=-H_z. \tag{3}$$

Here, φ is the DW angle. This equation is split into left and right sides with H_x because H_x generate opposite $H_{\parallel}$ at each side. The first order of approximation equation at each side of the facet is:

$$H_{\mathrm{SMT}}\cos\varphi_{\mathrm{L}}\,/\left[1+\left(\frac{\pi}{2}\right)\frac{H_x\sin\varphi_{\mathrm{L}}}{H_K}\right]\cong -H_z, \tag{4a}$$

$$H_{\mathrm{SMT}}\cos\varphi_{\mathrm{R}}\,/\left[1-\left(\frac{\pi}{2}\right)\frac{H_x\sin\varphi_{\mathrm{R}}}{H_K}\right]\cong -H_z. \tag{4b}$$

The left side of above two equations should have the same value; thus,

$$\left[1+\left(\frac{\pi}{2}\right)\frac{H_x\sin\varphi_{\mathrm{L}}}{H_K}\right]\cos\varphi_{\mathrm{R}}\cong\left[1-\left(\frac{\pi}{2}\right)\frac{H_x\sin\varphi_{\mathrm{R}}}{H_K}\right]\cos\varphi_{\mathrm{L}}. \tag{5}$$

Using trigonometric identities, we obtain the following SMT-only facet tilting equation:

$$\frac{\sin\Phi_-}{\cos\Phi_+}\cong\left(\frac{\pi}{2}\right)\frac{H_x}{H_K}. \tag{6}$$

Here, $\Phi_+=(\varphi_{\mathrm{R}}+\varphi_{\mathrm{L}})/2$ and $\Phi_-=(\varphi_{\mathrm{R}}-\varphi_{\mathrm{L}})/2$.

Recent research on the SMT shows that the change of the SMT is similar to the change of the wall. But the variation of SMT is much more than expected from the wall width changes. They explained the origin is that the β depends on the wall width[16-18]. Thus, we replace H_K with H_K^* in Supplementary Equation (6) as follows.

$$\frac{\sin\Phi_-}{\cos\Phi_+}\cong\left(\frac{\pi}{2}\right)\frac{H_x}{H_K^*}. \tag{7}$$

Here, H_K^* is an effective anisotropy representing effects of the wall width variation as well as β variation.

SOT-induced facet tilting has to consider the DW magnetization. Without H_x, the DW magnetization angle (ψ) is stabilized by H_{SOT}:

$$H_{\mathrm{SOT}} \cos \psi = -H_z. \tag{8}$$

Due to H_{DMI}, ψ should have the same value as φ. However, applied H_x breaks this situation. The projection field of H_x and H_{DMI} on the transverse direction of DW magnetization should balance out at each side of the facet as follows:

$$H_{\mathrm{DMI}} \sin(\varphi_{\mathrm{R}} - \psi) = H_x \cos \psi, \tag{9a}$$

$$H_{\mathrm{DMI}} \sin(\psi - \varphi_{\mathrm{L}}) = H_x \cos \psi\ . \tag{9b}$$

Elimination of ψ leaves:

$$\frac{\sin \Phi_-}{\cos \Phi_+} = \frac{H_x}{H_{\mathrm{DMI}}}. \tag{10}$$

This is the SMT-only facet-tilting equation.

Note that these tilting equations are quite simplified. The adiabatic SMT and the field-like SOT act as additional in-plane fields that can make an offset in the facet-tilting equation. However, in our experiments, we observed no significant offsets in the facet-tilting experiments (significant offsets are observed in other samples, discuss later). We believe that the induced field by the adiabatic SMT and the field-like SOT is negligibly small compared with H_x.

Finally, if we ignore the offset of tilting and we measure small change of Φ_-, replacing $\sin\Phi_-$ with $\Delta\Phi_-$ make a more simple equation used in the main text. The difference between sin(x) and x is not larger than 5 % up to x=0.5.

Supplementary Note 4

: Facet sharpening

Similar to the facet-tilting equation, we can derive the facet-sharpening equation. The sharpening does not distinguish left and right sides of the facet. Thus, we only consider the left side of the facet. In the case of the SMT-only facet, applied H_y expands the DW width and then the facet angle equation is:

$$H_{\mathrm{SMT}} \cos \varphi_{\mathrm{L}} \Big/ \left[1+\left(\frac{\pi}{2}\right)\frac{H_y \cos \varphi_{\mathrm{L}}}{H_K{}^*}\right] \cong -H_z. \tag{11}$$

We use $H_K{}^*$ than H_K to include the effect of β variation on the wall width[16-18]. We divided Supplementary Equations (11) by Supplementary Equation (3) and replaced φ_{L} (φ) with Φ_+ (Φ_{+0}). Then,

$$\frac{1}{\cos \Phi_+} - \frac{1}{\cos \Phi_{+0}} \cong -\left(\frac{\pi}{2}\right)\frac{H_y}{H_K{}^*}. \tag{12}$$

This is the facet-sharpening equation of SMT only case. In addition, assuming $|\Phi_+ - \Phi_{+0}| \ll 1$ results in following asymptotic equation.

$$\frac{\sin(\Phi_+ - \Phi_{+0})}{\sin \Phi_{+0}} \cong -\left(\frac{\pi}{2\tan^2 \Phi_{+0}}\right)\frac{H_y}{H_K{}^*}. \tag{13}$$

It is notable that facet observation is easy with small H_z because larger H_z induces more nucleation in the samples that erases the facet domains. Thus, most of observable facet has angle near $\Phi_+ \sim 90^\circ$ As a result, the effect of SMT in sharpening equation is much smaller than that of in the tilting equation.

The facet sharpening equation for SOT only case is also obtained. Projected fields of H_y and H_{DMI} on the transverse direction of DW magnetization should balance out that requires,

$$H_{\mathrm{DMI}} \sin(\varphi_{\mathrm{L}} - \psi) = H_y \sin \psi. \tag{14}$$

We replaced φ_{L} (φ) with Φ_+ (Φ_{+0}) then,

$$\frac{\sin(\Phi_+ - \Phi_{+0})}{\sin \Phi_{+0}} = \frac{H_y}{H_{\mathrm{DMI}}}. \tag{15}$$

This is the simplest facet sharpening equation of the SOT-only case that is useful when $H_K \gg H_{\mathrm{DMI}}$. If H_{DMI} is comparable with H_K, we have to consider the domain tilting effect. Applied H_y tilts the domain and makes m_y

component ~H_y/H_K with small H_y. This m_y component generates SOT fields with the amount of $\tau_d H_y/H_K$ in domains. But at the domain wall centre, there is only pure in-plane magnetization component thus ψ should be changed by following equation to produce the SOT field.

$$\cos\psi - C_1\frac{H_y}{H_K} = \cos\psi_0 = -\frac{H_z}{H_{\mathrm{SOT}}}. \quad (16)$$

Here, ψ_0 is the domain wall magnetization angle at H_y=0. C_1 is a constant and is expected within the range of 0~1. Micromagnetic simulations find 0.5 is good for C_1 (Supplementary Information 7). Combining Supplementary Equations (14) and (16) produce a correction factor to Supplementary Equation (15) as follows.

$$\frac{\sin(\Phi_+ - \Phi_{+0})}{\sin\Phi_{+0}} \cong \frac{H_y}{H_{\mathrm{DMI}}}\left(1 - \frac{C_2}{\sin^2\Phi_{+0}}\frac{|H_{\mathrm{DMI}}|}{H_K}\right). \quad (17)$$

C_2 is a constant almost equal to C_1. However, best value of C_2 is 0.8 obtained from micromagnetic simulations (Supplementary Note 7). We think this discrepancy comes from that Supplementary Equation (16) take into account only centre of the domain wall but Supplementary Equation (17) considers averaged SOT over the domain wall.

Finally, if we measure small change of Φ_+, replacing $\sin(\Phi_+ - \Phi_{+0})$ with $\Delta\Phi_+$ make a more simple equation used in the main text.

Supplementary Note 5

: Estimation of $\boldsymbol{H}_{\mathrm{SOT}}$ and $\boldsymbol{H}_{\mathrm{DMI}}$

We can obtain H_{SOT} and H_{DMI} by measuring offset fields of facets with In-plane magnetic field parallel to the current (for φ=±90°). In this state, the SMT field is zero. Also, the SOT field is zero due to sufficient H_{DMI}. Application of H_y (//I) makes this situation interesting. H_y tilts the domain wall magnetization from the DMI field direction to H_y that generate nonzero SOT field (~$H_{\mathrm{SOT}}\times H_y/|H_{\mathrm{DMI}}|$) with small H_y. However, the SMT field is still zero due to φ=±90°. This nonzero SOT field act as an offset field of facet angles in H_z. Thus, archiving proper H_z/H_y value for keeping φ=±90° results in H_z/H_y=–$H_{\mathrm{SOT}}/|H_{\mathrm{DMI}}|$. In experiments, we can get H_z/H_y by a linear fit of cosφ as a function of H_z under fixed H_y. The offset field ($H_{\mathrm{z,offset}}$) is the value of H_z for cosφ=0. Alternatively, finding a set of (H_y, $H_{\mathrm{z,offset}}$) for maintain φ=±90° sates with half-up and half-down magnetization sates is also possible. Including the domain tilting effect as a correction term, we get

$$\frac{H_{z,\mathrm{offset}}}{H_{\mathrm{SOT}}} \cong -\frac{H_y}{|H_{\mathrm{DMI}}|}\left(1 - C_2\frac{|H_{\mathrm{DMI}}|}{H_K}\right). \tag{18a}$$

This Supplementary Equation (18a) is useful for small H_y (<<H_{DMI}). Expanding H_y range up to large value (H_y >>H_{DMI}) results in

$$\frac{H_{z,\mathrm{offset}}}{H_{\mathrm{SOT}}} \cong -\frac{H_y}{\sqrt{{H_{\mathrm{DMI}}}^2 + {H_y}^2}}\left(1 - C_2\frac{|H_{\mathrm{DMI}}|}{H_K}\right). \tag{18b}$$

Using this equation, we also obtain each value of H_{SOT} and H_{DMI} because $H_{\mathrm{z,offset}}$ converges to ~H_{SOT} at large H_y. From the slope of $H_{\mathrm{z,offset}}/H_y$ near H_y=0, we can archive $|H_{\mathrm{DMI}}|$. Supplementary Figure 1 shows the results of $H_{\mathrm{z,offset}}$ as a function of H_y with positive and negative currents. We obtain H_{SOT}=–15.4±0.9 Oe and $|H_{\mathrm{DMI}}|$=1.25±0.12 kOe.

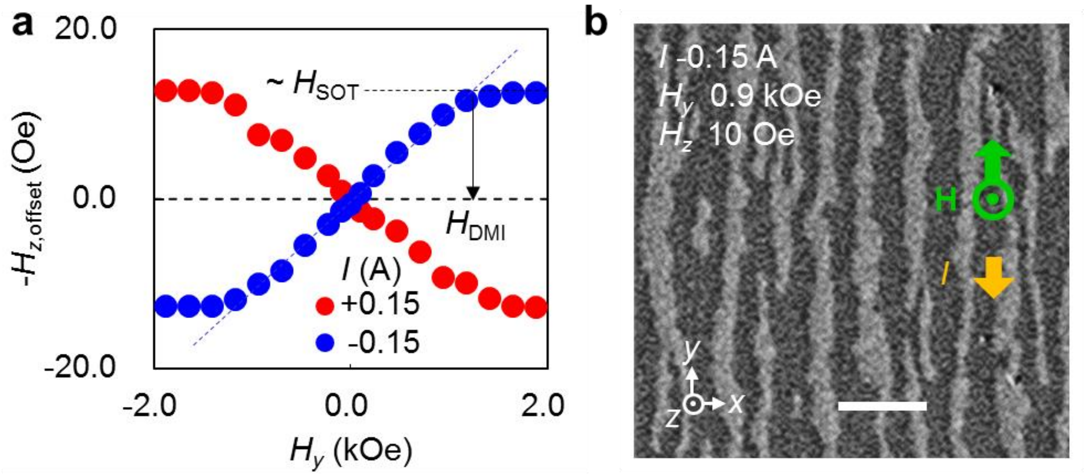

Supplementary Figure 1 | Measurement of $H_{z,offset}$. a, $H_{z,offset}$ as a function of H_y with opposite current directions. I=+0.15 A (red circles) and I=−0.15 A (blue circles). The positive current is parallel to the positive H_y. **b,** An example of parallel domain state. The scale bar is 10 μm.

Supplementary Note 6

: Estimation of H_K

We attempted to obtain H_K without changing I because different I not only breaks the field alignment but also changes the sample temperature. The magneto-optical Kerr signal is good for detecting the perpendicular magnetization component of the sample. Magnetic hysteresis loops are obtained from the image brightness. Supplementary Figure 2a shows hysteresis loops of the sample under different applied H_x and fixed I (0.15 A). The loops are normalized by the amplitude of $H_x = 0$ loop. These loops have saturated signals when H_z is sufficiently large ($|H_z| > 40$ Oe). Supplementary Figure 2b represents the saturated signal intensity as a function of H_x. We know the domain magnetization angle (θ) from the +z axis is as follows:

$$\cos\theta = \frac{H_K \cos\theta + H_z}{\sqrt{(H_K \cos\theta + H_z)^2 + H_x^2}}. \tag{19}$$

Fitting of the saturated MOKE signal by Supplementary Equation (19) estimates H_K (=6.1±0.3 kOe).

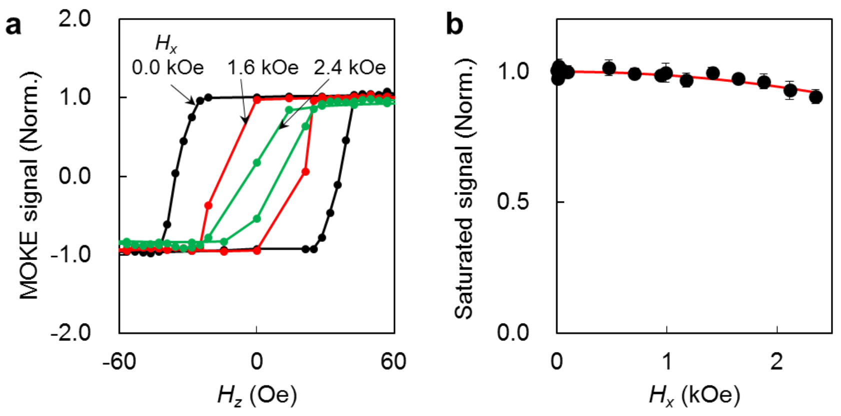

Supplementary Figure 2 | Estimation of H_k. a, Normalized MOKE hysteresis loop. **b,** Saturated MOKE signal as a function of H_x. The red line is a fitting line. Error bars are the standard deviation of ten measurements.

Supplementary Note 7

: Micromagnetic simulations for SOT- and SMT-only facet

Micromagnetic simulations show the validity of the facet tilting and the facet sharpening. The simulation geometry is described in Method section. The basic material parameters are as follows. The saturation magnetization (M_S) is 900×10^3 A m^{-1}, the exchange stiffness constant (A) is 1×10^{-11} J m^{-1}, the anisotropy constant (K) is 0.8×10^6 J m^{-3}, and the interfacial Dzyaloshinskii–Moriya interaction (D) is –1 mJ m^{-2}.

Supplementary Figure 3 shows the tilting and the sharpening of the SOT-only facet with different parameter sets. Supplementary Equation (10) means an exact linear relation between $\sin(\Phi_-)/\cos(\Phi_+)$ and H_x/H_{DMI}. This linear relation is shown in Supplementary Fig. 3a. The sharpening results (Supplementary Fig. 3b) also show a clear linear relation of $\sin(\Phi_+-\Phi_{+0})/\sin(\Phi_{+0})$ on H_y with some correction terms, as described by Supplementary Equation (17). From the sharpening simulation, we obtained C_2=0.8 for Supplementary Equation (17). The upper inset of Supplementary Fig. 3b shows the magnetization angle at the domain wall centre (ψ). The linear line comes from Supplementary Equation (16), with C_1=0.5. Note that we used H_{DMI}^* $(=D/(\varDelta_0 M_S))$, $\varDelta_0$ $(=(A/K_{eff})^{1/2}$, K_{eff} $(=K-(\mu_0/2)M_S^2)$, and H_K $(=2K_{eff}/M_S)$. We know that the domain wall magnetization is fixed by H_{DMI} $(=H_{DMI}^*-H_S)$, where H_S $(\approx 2\ln 2\mu_0 M_S t_f/(\pi^2\varDelta_0))$ is the demagnetization field of the Néel wall and t_f is the thickness of the magnetic layer.

Supplementary Figure 4 represents the simulation results of the SMT-only facet with different parameter sets. Tilting of facets (Supplementary Fig. 4a) shows a good linear relation between $\sin(\Phi_-)/\cos(\Phi_+)$ and $(\pi/2)H_x/H_K$, as expected by Supplementary Equation (6), but the results of sharpening show a significant quadratic dependence of $\sin(\Phi_+-\Phi_{+0})/\sin(\Phi_{+0})$ on H_y (Supplementary Fig. 4b). Despite these quadratic dependences, the curves have a tangent line with a slope near H_y=0, as expected by Supplementary Equation (13). It is therefore useful for obtaining the linear relation of sharpening that derives an odd function such as $f_{odd}(+H_y)=\{f(+H_y)-f(-H_y)\}/2$. The inset of Supplementary Fig. 4b exhibits the odd function of $\sin(\Phi_+-\Phi_{+0})/\sin(\Phi_{+0})$ showing a linear relation near H_y=0, as expected by Supplementary Equation (13).

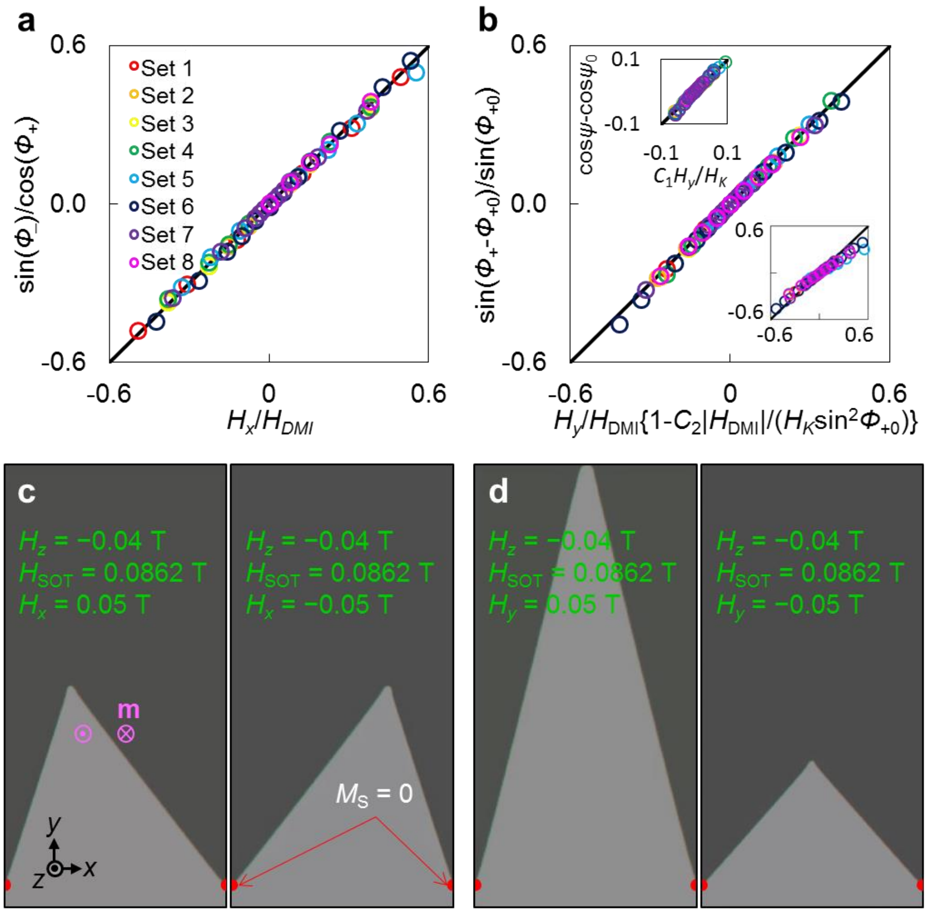

Supplementary Figure 3 | Micromagnetic simulations for SOT-only facets. a, Tilting results of the facet as a function of H_x with different material parameters. The black solid line is an exact linear relation. **b,** Sharpening results of the facet as a function of H_y. Upper inset shows the variation of in the magnetization angle at the domain wall centre. Here, C_1 is 0.5 and C_2 is 0.8. Lower inset shows the results with C_2=0. **c,** Examples of tilting from set 3. **d,** Examples of sharpening from set 3. Each set has parameters that differ from the basic values as follows. Set 1: H_{SOT} (0.115 T), H_z (–0.04 T). Set 2: H_{SOT} (0.0862 T), H_z (–0.03 T), K (0.7×10^5 J m^{-3}). Set 3: H_{SOT} (0.0862 T), H_z (–0.04 T), K (0.7×10^5 J m^{-3}). Set 4: H_{SOT} (0.0862 T), H_z (–0.05 T), K (0.7×10^5 J m^{-3}). Set 5: H_{SOT} (0.0862 T), H_z (–0.04 T), K (0.6×10^5 J m^{-3}). Set 6: H_{SOT} (0.0862 T), H_z (–0.04 T), K (0.9×10^5 J m^{-3}). Set 7: H_{SOT} (0.0862 T), H_z (–0.04 T), K (0.7×10^5 J m^{-3}), D (–0.5 mJ m^{-2}). Set 8: H_{SOT} (0.0431 T), H_z (–0.02 T), K (0.7×10^5 J m^{-3}).

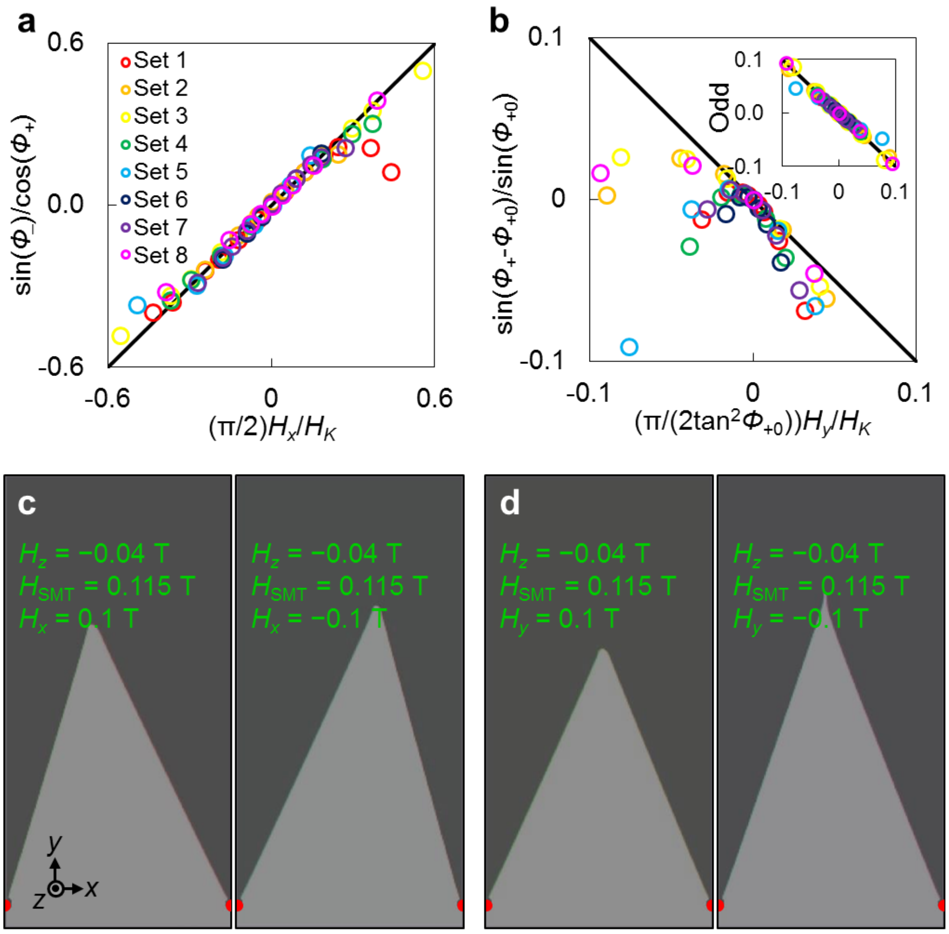

Supplementary Figure 4 | Micromagnetic simulations for SMT-only facets. a, Tilting results. The black solid line is an exact linear relation. **b,** Sharpening results. Inset shows the odd function of $\sin(\Phi_{+}-\Phi_{+0})/\sin(\Phi_{+0})$. **c,** Examples of tilting from set 1. **d,** Examples of sharpening from set 1. The parameters of each set have changed. Set 1: H_{SMT} (0.115 T), H_z (–0.04 T). Set 2: H_{SMT} (0.115 T), H_z (–0.06 T). Set 3: H_{SMT} (0.089 T), H_z (–0.04 T), K (0.7×10^5 J m^{-3}), D (–1.5 mJ m^{-2}). Set 4: H_{SMT} (0.089 T), H_z (–0.03 T), K (0.7×10^5 J m^{-3}). Set 5: H_{SMT} (0.089 T), H_z (–0.04 T), K (0.7×10^5 J m^{-3}), D (–0.7 mJ m^{-2}). Set 6: H_{SMT} (0.064 T), H_z (–0.02 T), K (0.9×10^5 J m^{-3}). Set 7: H_{SMT} (0.128 T), H_z (–0.05 T), K (0.9×10^5 J m^{-3}). Set 8: H_{SMT} (0.062 T), H_z (–0.03 T), K (0.6×10^5 J m^{-3}).

Supplementary Note 8

: Temperature and the Oersted field problem

The electric current needed to generate sufficient spin-torque effects requires current density larger than 10^9~10^{10} A m^{-2}. Thus, flowing such current density in the film structure (~1 mm in width, ~10 nm in thickness) requires several hundred mA of total current. This current heats up the sample as well as the sample stage. The stabilized temperature should be higher than room temperature. For example, film that is 3 mm in width and 0.6 mm in length shows clear facet domains with 0.29 A of total current, but with a sample temperature of ~370 K. This current is sufficient for changing the material parameters.

To remove this heating effect, it is good to reduce the sample size to decrease the total current; however, the small size should generate significant Oersted field distribution. Typically, current density ~10^{10} A m^{-2}, sample width ~1 mm, and total thickness ~10 nm induces a perpendicular field gradient ~1 Oe mm^{-1} along the sample width near the sample centre. If the Oersted field gradient is comparable to the spin-torque fields, we can see gradual angle variation of the facets. To reduce this problem, it is advantageous to increase the sample width.

Therefore, we have to tune the sample size. In this paper, we select a sample size of 1 mm in width and 0.2 mm in length. This sample shows facet formation with 0.15 A of current. The total current becomes almost half that of film 3 mm in width and 0.6 mm in length, so the temperature increase is reduced by ~1/4. As a result, we performed all experiments under the sample temperature of ~320 K. We think that this temperature increase does not induce a significant difference from room temperature (see the next section) Also, we only observe a small area (<100 μm×100 μm) near the centre of the film to minimize the Oersted field effect (<0.1 Oe).

Note that the Oersted field also has a transverse component (*x*-directional field by *y*-directional current) which does not exceed several tens of Oersted by ~10^{10} A m^{-2} current density. However, the magnetic layer is placed in almost the centre of the film stack that cancels the transverse component of the Oersted field. Moreover, tilting and sharpening are observed with the ~kOe in-plane field. Therefore, the in-plane component of the Oersted field is negligible in our experiments.

Supplementary Note 9

: Comparison with other methods

We check our results through a comparison with other methods. In the wire structure, magnetic field-induced domain wall motions with small electric current are a well-known method to extract the pure current effect[16]. The domain wall speed is mainly determined by the magnetic field, but the current makes a small speed deviation. We fabricate a wire 30 μm in width and select the current density to be 7.1×10^9 A m^{-2}. The total current is sufficiently small and does not induce a meaningful temperature increase (<2 K)[16]. Supplementary Figure 5a shows the wire structure and measured domain wall speeds with $+I$ and $-I$ and several H_z. If we shift oppositely, H_z by H_{shift} (pink arrows) depend on the current direction that collects the speeds in a single curve because opposite currents generate opposite spin-torque effects. H_{shift} corresponds to H_{ST}. Dividing H_{ST} by the current density provides the spin-torque efficiency (ε). Supplementary Figure 5b shows ε as a function of H_y. For simplicity, we assume a uniform current density in the all stack except for MgO layer. When H_y is large, the magnetization exhibits nucleation-dominant behaviour as opposed to the wall motion that interrupts the measurement of ε. We perform the same experiments with different sample temperatures using a heater attached in the sample stage. The obtained ε does not significantly change up to 340 K. As the facet experiments in the paper are done at ~320 K, we think that an increase in temperature by 20 K is allowable to estimate the room temperature (~300 K) value of spin torques.

Note that if we know H_{DMI}^*, we can separate $\varepsilon_{\mathrm{SOT}}$ and $\varepsilon_{\mathrm{SMT}}$ from ε. The ε subscript denotes each kind of spin torque. The experiments for the asymmetric expansion of circular domains[12] determine that $H_{\mathrm{DMI}}^* = 0.78\pm0.05$ kOe, but several studies have shown other origins of asymmetric expansion[19]. Therefore, we used only the circular domain expansion to determine the sign of H_{DMI}. In addition, our experiments determine that $H_{\mathrm{DMI}}=1.25\pm0.12$ kOe, which means that $H_{\mathrm{DMI}}^*(=H_{\mathrm{DMI}}+H_{\mathrm{S}})$ is larger than 1.25 kOe; thus, separation of H_{SOT} and H_{SMT} is impossible from the wire experiments.

However, we can compare the facet experiments with the wire experiments. The value of $\varepsilon_{\mathrm{ST}}$ with zero in-plane field obtained from the facet experiments, shown as a black solid line in Supplementary Fig. 5b, and the value well fit the wire experiments at $H_y=0$. In addition, the facet experiments estimate the linear slope of each spin torque on H_y near $H_y=0$. $H_{\mathrm{SMT}}(1-(\pi/2)(H_y/H_K^*))$ is the first-order approximation of the SMT field. Tilting of the magnetic domain slightly reduces H_{SOT} such as $H_{\mathrm{SOT}}(1-C_1(H_y/H_K))$. We plot these dependences by converting

each spin-torque field to each ε in Supplementary Fig. 5b. Then, ε_{ST} (=ε_{SOT}+ε_{SMT}) has a similar slope on H_y with measured ε in the wire.

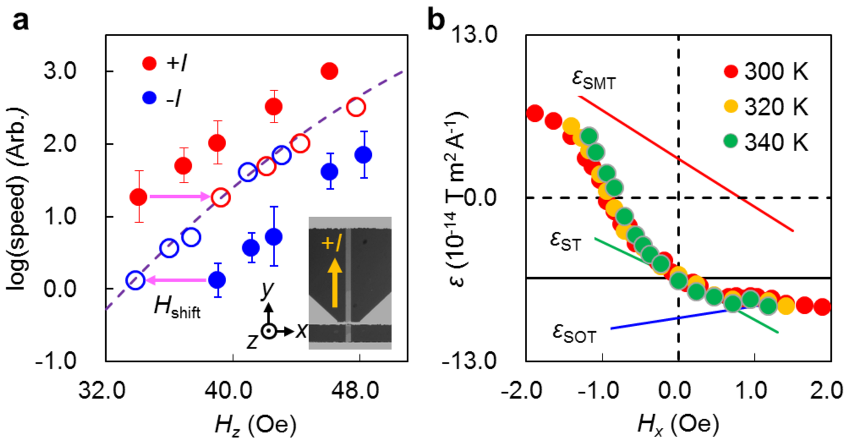

Supplementary Figure 5 | Spin torques measured in wire structures. a, Domain wall speeds with respect to H_z. Positive and negative currents generate speed differences. Opposite field shifts (H_{shift}, pink arrows) in H_z merge the wall speeds into a single curve. The inset shows the wire structure. Error bars are the standard deviation of ten measurements. **b,** The spin-torque efficiency (ε) as a function of H_y. Black solid line is the expected value from the facet experiments. Red, green, and blue solid lines represent the linear slope of each spin-torque efficiency expected from the facet experiments.

Supplementary Note 10

: Other sample 1: Sub/Ta(3 nm)/Pt(3 nm)/CoFeB(0.9 nm)/Pt(0.6 nm)/MgO(1.5 nm)/Ta(2 nm)/Pt(1.5 nm)

The sample has a thicker insertion Pt layer (0.6 nm) than that of the sample in the main script (0.4 nm). The film is patterned to 1 mm in width and 0.2 mm in length. The sample has an additional capping layer of Pt 1.5 nm to improve electrode contacts, which drastically reduces the total resistance. As a result, 0.3 A of current shows clear facet domains at a sample temperature of ~320 K.

The film shows perpendicular magnetization and stripe growth behaviour. Such stripe growth shows strong asymmetry in domain growth. Supplementary Figure 6a–c shows the typical stripe growth. We saturate the magnetization in the –*z* direction and apply 10 Oe of H_z. Next, the stripe domains grow from the nucleation site up to 20 s. After growth, –10 Oe of H_z are applied to compress the expanded domain. However, the boundary of the stripe domain does not go back to the initial nucleation site, and only the relative proportion of +*z* and –*z* magnetization are changed in the striped domain (insets of Supplementary Fig. 6b,c). Therefore, we can expect that there is a magnetic field on the boundary of the striped domain for converting the uniform magnetization to the striped domain state. Here, we call this field a stripe field, H_{stripe}. The insets of Supplementary Fig. 6a schematically show where H_{stripe} acts as a force and the direction of the field. We think that the main cause of H_{stripe} is the uncompensated dipolar field generated from the domain magnetizations.

Supplementary Figure 6d exhibits the facet angles under +0.3 A of *I* with respect to H_z. The red line is the expected trend determined by independent experiments of field-driven motions assisted by electric current[16]. At a large field of $|H_z|$ (>4 Oe), $\cos(\Phi_+)$ converges to the linear line, but small H_z makes significant deviations from the linear line. Moreover, even a negative H_z makes facet domains in the –*z* initial magnetization state. We think that the origin of such deviations from a simple linear line is H_{stripe} because a larger H_z squeezes the striped domain and makes it almost uniform near the facet boundary (see the green boxed area in the insets of Supplementary Fig. 6d), resulting in $\cos(\Phi_+)$ following the simple linear relation on H_z. If we find the difference of applied H_z and the linear line, we obtain H_{stripe} as a function of H_z (Supplementary Fig. 6e). Beside this H_{stripe}, the stripe growth-dominant sample is also useful for facet experiments. A large H_z for erasing the stripe state near the facet boundary clearly measures the total spin-torque fields (H_{ST}=–10.8±0.4 Oe).

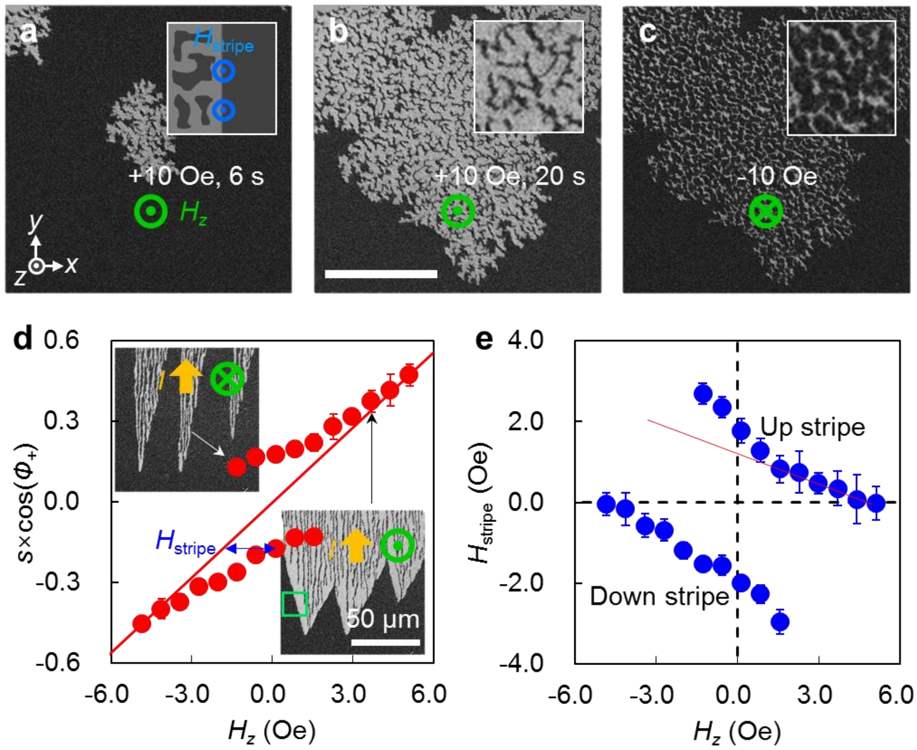

Supplementary Figure 6 | Stripe domains and facet experiments with Sub/Ta(3 nm)/Pt(3 nm)/CoFeB(0.9 nm)/Pt(0.6 nm)/MgO(1.5 nm)/Ta(2 nm)/Pt(1.5 nm) sample. a–c, Growth and compression of stripe domains. The boundary of the stripe domain is conserved under compression. Inset of **a** shows the stripe field H_{stripe}. Insets of **b** and **c** show magnified images of the stripe domains. The scale bar is 50 μm. **d,** cos(Φ_+) as a function of H_z under +0.3 A of I. Inset images show facets under conditions indicated by black and white arrows. **e,** Estimations of H_{stripe} as a function of H_z. Error bars are the standard deviation of five measurements.

We can also observe tilting and sharpening of facets in this sample. Supplementary Figure 7 shows the results. Before facet experiments, we obtained H_K=2.2±0.1 kOe with 0.3 A of I. Although I increases the sample temperature (~320 K), H_K is not much different from the room temperature value (H_K=2.3±0.1 kOe). Therefore, we expect that other magnetic parameters will not change much from room temperature. Supplementary Figure 7a shows $H_{z,\mathrm{offset}}$ with respect to ±H_y and ±I. Using this and the obtained H_{ST}, we get H_{SOT}=–15.4±0.8 Oe, H_{SMT}=+4.7±1.2 Oe, and H_{DMI}=0.47±0.03 kOe. Supplementary Figure 7b exhibits several examples of facets, with each diamond symbol indicating the magnetic field conditions in Supplementary Fig. 7c and d. Supplementary Figure 7c shows tilting of facets with +H_z. The results show a positive slope on H_x, which means

that the SOT effect is dominant in the facet tilting. From the linear fitting of the results, we obtain H_K*=0.24±0.08 kOe, which is much smaller than H_K owing to the β dependence on the wall width[16].

It is notable that the tilting results have a significant offset in H_x (~0.45 kOe). The current generates the in-plane Oersted field which does not exceed tens of Oe on the film surface. In addition, the magnetic layers are placed near the centre of the stack structure, which reduces the Oersted field effect. The field-like SOT acts as an effective H_x in the facet tilting equation. However, if we roughly estimate the strength of the field-like SOT to be similar to H_{SOT}, this does not explain the offset. Therefore, we think that the SOT is affected by the adiabatic SMT, which induces the magnetization rotation in the domain walls, thereby generating an additional SOT field. This effect is shown in Fig. 5.

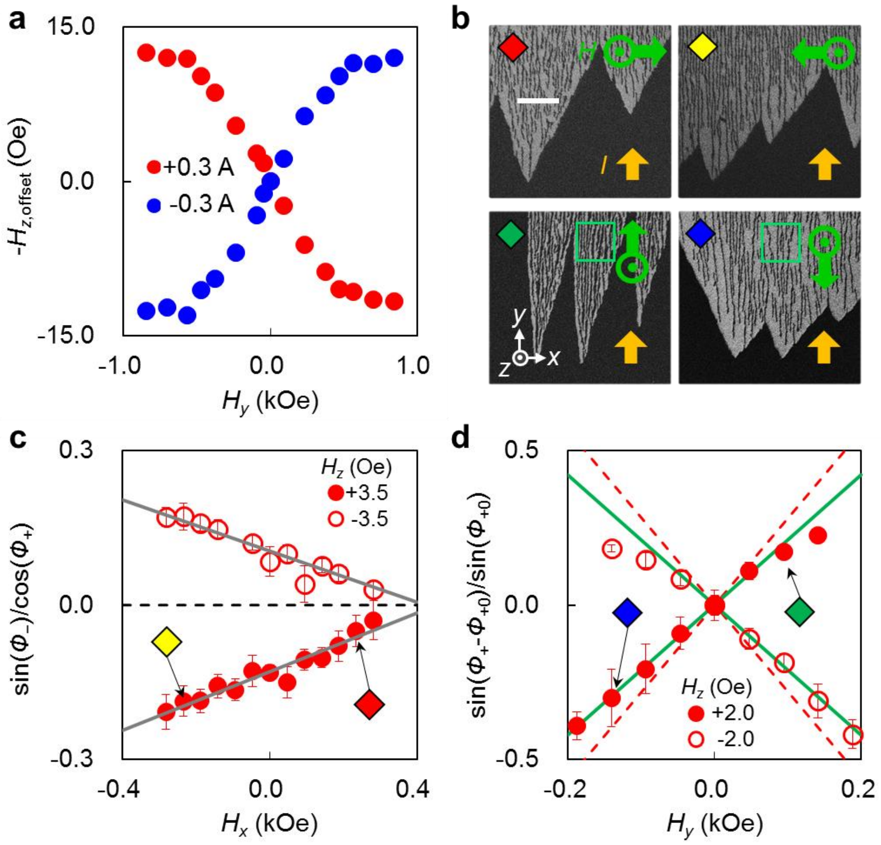

Supplementary Figure 7 | Facet experiment with Sub/Ta(3 nm)/Pt(3 nm)/CoFeB(0.9 nm)/Pt(0.6 nm)/MgO(1.5 nm)/Ta(2 nm)/Pt(1.5 nm) sample. a, $H_{z,offset}$ with respect to H_y and I. **b,** Facets with different conditions. Diamond symbols indicate conditions in **c** and **d**. The scale bar is 20 μm. **c,** Tilting of facet with +0.3 A of I. **d,** Sharpening of facets with +0.3 A of I. Error bars are the standard deviation of five measurements.

Using the obtained parameters, we can estimate the slope of facet sharpening. Supplementary Figure 7d shows the sharpening result and expected slope of sharpening as red dashed lines from the obtained H_{SOT}, H_{SMT}, H_{DMI}, H_K, and H_K^{*}. The dashed lines are similar to the experimental results, but with a small discrepancy. We assumed that the change in H_{stripe} was a major cause of this difference. The green boxes in Supplementary Fig. 7b show the relative portion of +*z* and –*z* magnetization in the stripe domain under the same H_z. Such relative portion variation is explained by Supplementary Equation (18a) because the stripe domain has parallel alignment with the current direction. $H_{z,\mathrm{offset}}$ acts as an effective H_z in the stripe domain that changes the relative portion of +z and –z states. Such portion change induces the variation in H_{stripe}. Including the H_{stripe} effect in the sharpening equation results in the following.

$$\frac{\sin(\Phi_{+}-\Phi_{+0})}{\sin\Phi_{+0}} \cong \left[\frac{H_{\mathrm{SOT}}}{H_{\mathrm{DMI}}}\left(1-\frac{C_2|H_{\mathrm{DMI}}|}{H_K\sin^2\Phi_{+0}}\right)-\frac{\pi H_{\mathrm{SOT}}}{2H_K^{*}\tan^2\Phi_{+0}}+\frac{R_{sz}H_{\mathrm{SOT}}}{H_{\mathrm{DMI}}\sin^2\Phi_{+0}}\left(1-\frac{C_2|H_{\mathrm{DMI}}|}{H_K}\right)\right]\frac{H_y}{H_{\mathrm{ST}}}. \quad (20)$$

Here, R_{sz} (~ –0.25) is the variation rate of H_{stripe} on H_z near H_z=2.0 Oe, which is depicted as a red line in Supplementary Fig. 6e. The estimation of the facet sharpening from this equation shows good accordance with the experiments (green lines in Supplementary Fig. 7d).

Supplementary Note 11

: Other sample 2: Sub/Ta(3 nm)/Pt(3 nm)/CoFeB(0.9 nm)/MgO(1.5 nm)/Ta(2 nm)

We also perform facet experiments with Sub/Ta(3 nm)/Pt(3 nm)/CoFeB(0.9 nm)/MgO(1.5 nm)/Ta(2 nm) samples. The samples are patterned to 0.16 mm in width and 0.6 mm in length, and 0.01 A of current easily makes facet domains with temperature increases less than 5 K. The measured H_K is 6.4±0.2 kOe with or without the current (0.01A). This film prefers the stripe domain state (Supplementary Fig. 8a), so we independently measure the field-driven wall motions assisted by electric current in the same sample. Supplementary Figure 8b and c shows examples of the facet. Diamond symbols indicate the applied field in Supplementary Fig. 8d, which compares the facet data, and the red line represents the expectation from the field-driven wall motion experiments. The obtained H_{ST} is -8.3±0.7 Oe. Supplementary Figure 8e shows an estimation of H_{stripe}.

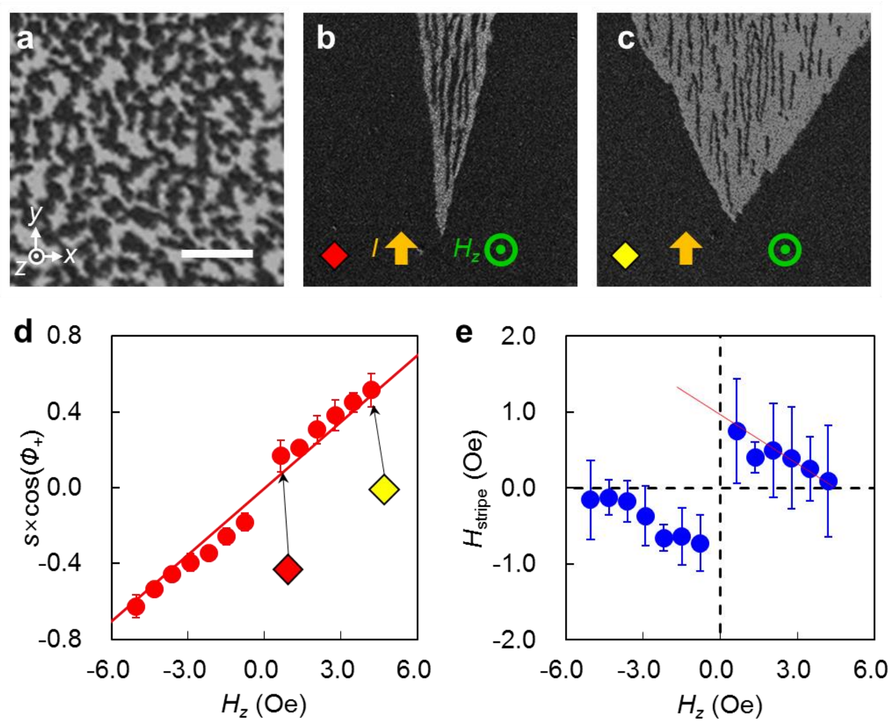

Supplementary Figure 8 | Stripe domains and facet experiments with Sub/Ta(3 nm)/Pt(3 nm)/CoFeB(0.9 nm)/MgO(1.5 nm)/Ta(2 nm) sample. a, Stripe domain state of the sample. The scale bar is 20 µm. **b,c** examples of facets with I=+0.01. Diamond symbols indicate the experimental conditions in **d**. **d,** cosΦ_+ as a function of H_z with I=+0.01. **e,** Estimation of H_{stripe}. Error bars are the standard deviation of five measurements.

Supplementary Figure 9a shows $H_{z,\mathrm{offset}}$ with respect to I and H_y. The film has a large H_{DMI} that exceeds our measurement field range (3 kOe). Thus, we only get the ratio $H_{\mathrm{SOT}}/H_{\mathrm{DMI}}[1–C_2(|H_{\mathrm{DMI}}|/H_K)]$, meaning that the facet experiments only detect the relative contributions of $H_{\mathrm{SOT}}/H_{\mathrm{DMI}}$ and H_{SMT}/H_K^* on the facet equations. Nevertheless, the results are interesting because the facet tilting is not explained by the SOT effect. Supplementary Figure 9c shows the tilting results of the facets. The applied $+I$ and $+ H_z$ should make a positive slope from the SOT effect, but the results exhibit a negative slope, which implies that the amount of $(\pi/2)H_{\mathrm{SMT}}/H_K^*$ overcomes $H_{\mathrm{SOT}}/H_{\mathrm{DMI}}$ and that H_{SMT} is meaningful. If H_{DMI} has a value between 3 kOe and 5 kOe, H_{SMT}/H_K^* is –2.0 ~ –1.5 times larger than $H_{\mathrm{SOT}}/H_{\mathrm{DMI}}$. We think that a large H_{DMI} sufficiently reduces the $H_{\mathrm{SOT}}/H_{\mathrm{DMI}}$ term, but that a large H_K increases the H_{SMT}. Despite the increase in the relative value of H_{SMT}/H_K^*, the sharpening of facet data shows a positive slope for $+I$ and $+H_z$ because the factor of $1/\tan^2\Phi_0$ is ~0.13 in the SMT sharpening equation (Supplementary Fig. 9d). Note that, from the $H_{z,\mathrm{offset}}$ measurements, facet tilting experiments, and H_{stripe}, we can estimate the slope of sharpening of the facet, which is shown as green lines in Supplementary Fig. 9d.

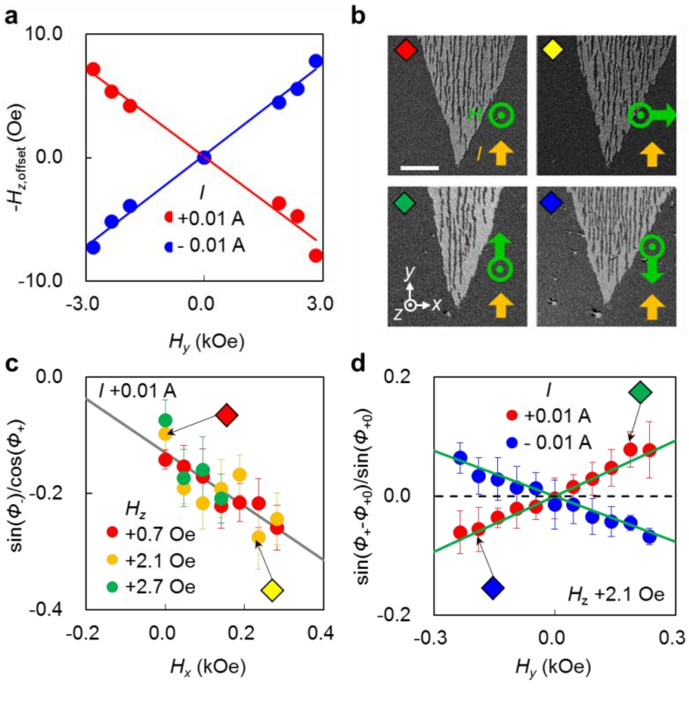

Supplementary Figure 9 | Facet experiment with Sub/Ta(3 nm)/Pt(3 nm)/CoFeB(0.9 nm)/MgO(1.5 nm)/Ta(2 nm) sample. a, $H_{z,\mathrm{offset}}$ with respect to H_y and I. **b,** Facets with different conditions. Diamond symbols indicate the current and field conditions in **c** and **d**. The scale bar is 20 μm. **c,** Tilting of facet. **d,** Sharpening of facets. Error bars are the standard deviation of five measurements.

For this sample, we estimate each spin-torque field based on our previous experiments. Fortunately, this film (Sub/Ta/Pt/CoFeB/MgO/Ta) has an H_k value and CoFeB thickness similar to the sample used in the main script (Sub/Ta/Pt/CoFeB/Pt(0.4 nm)/MgO/Ta); therefore, we extrapolate the H_{SMT} from Sub/Ta/Pt/CoFeB/Pt(0.4 nm)/MgO/Ta sample. If we employ a crude assumption that other material parameters are the same and only H_k is different, then the wall width of Sub/Ta/Pt/CoFeB/MgO/Ta should be ~97% that of the Sub/Ta/Pt/CoFeB/Pt(0.4 nm)/MgO/Ta sample. In the Sub/Ta/Pt/CoFeB/Pt(0.4 nm)/MgO/Ta sample, the change of H_{SMT} is ~4.8 times larger than the wall width change. Using this crude assumption, we can estimate that the ε_{SMT} of Sub/Ta/Pt/CoFeB/MgO/Ta is ~1.14 times larger than that of Sub/Ta/Pt/CoFeB/Pt(0.4 nm)/MgO/Ta.

Finally, we plot the spin-torque efficiencies of three different samples, (Sub/Ta(3)/Pt(3)/CoFeB(0.9)/Pt(0.0)/MgO(1.5)/Ta(2), thickness in nm), (Sub/Ta(3)/Pt(3)/CoFeB(0.9)/Pt(0.4)/MgO(1.5)/Ta(2), thickness in nm), and (Sub/Ta(3)/Pt(3)/CoFeB(0.9)/Pt(0.6)/MgO(1.5)/Ta(2)/Pt(1.5), thickness in nm), as a function of the inserted Pt thickness between CoFeB and MgO. Supplementary Figure 10 shows the results. Here, the $\varepsilon_{\mathrm{SOT}}$ and $\varepsilon_{\mathrm{SMT}}$ of the Pt 0.0 nm sample are extrapolated values. For simplicity, we assume a uniform current density in the all stack except for MgO layer.

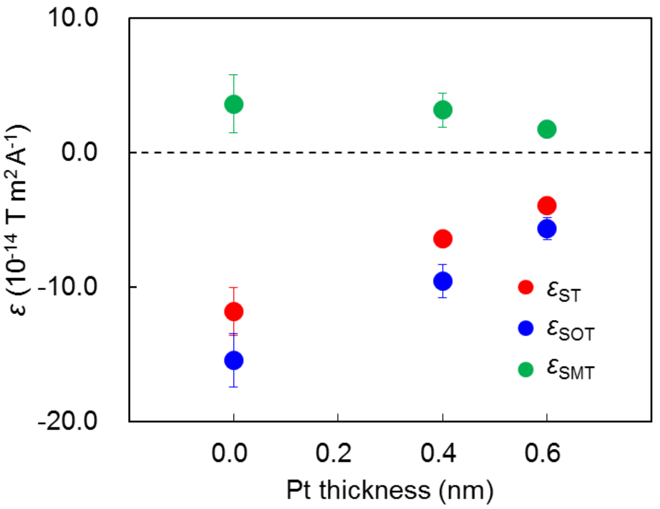

Supplementary Figure 10 | Spin-torque efficiencies. Values of 0.4 nm and 0.6 nm are obtained by facet experiments. The $\varepsilon_{\mathrm{SOT}}$ and $\varepsilon_{\mathrm{SMT}}$ of 0.0 nm are extrapolated values from 0.4 nm data.

Supplementary Note 12

: Weak DMI case

When H_{DMI} is smaller than H_S (demagnetization field of the Néel wall), the domain wall magnetization angle (from the current direction, ψ) should have two stable values at the same domain wall, such as $\psi = \varphi \pm \chi$ (solid and dashed pink arrows in Supplementary Fig. 11), where χ is $\cos^{-1}(H_{DMI}^*/H_S)$ and these two magnetization angles are energetically equivalent without H_x and H_y. Thus, we assume that the domain wall contains an equal proportion of magnetization with angles $\varphi + \chi$ and $\varphi - \chi$. Under this assumption, the SOT field ($H_{SOT,0}$) under zero H_x and H_y requires a correction factor $\cos\chi$ such as $H_{SOT,0}$=$\cos\chi$ $H_{SOT}(\psi$=0) because ψ is not aligned in the H_{DMI} direction. Regardless of this correction factor, the form of Equation (1) is not changed by using modified $H_{ST,0}$; $H_{ST,0}$=$H_{SOT,0}$+$H_{SMT,0}$. Here, $H_{ST,0}$ is the averaged spin-torque field on DW with zero H_x and H_y. Similarly, $H_{SMT,0}$ is the SMT field at a zero in-plane field with φ=0. Introducing $H_{SMT,0}$ does not matter because φ is the origin of the angular dependence of the SMT field. We think that this $H_{ST,0}$ is more important than $H_{ST}(\psi$=0) on DW motions because $H_{ST,0}$ induces the coherent unidirectional motion of multiple DWs under zero external magnetic field.

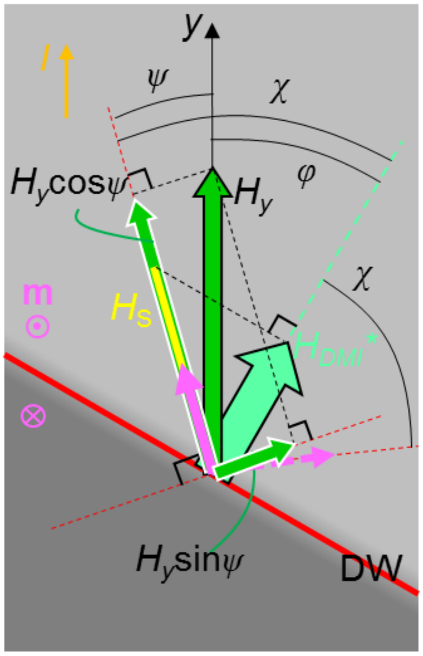

Supplementary Figure 11 | Magnetization angle of a weak DMI case. Solid and dashed pink arrows are two possible magnetizations of the domain wall.

The application of a nonzero in-plane field (H_x and H_y) complicates the problem because the in-plane fields tilt the DW magnetization angles. In addition, both the field and the thermal activation should change the relative portion of $\varphi + \chi$ and $\varphi - \chi$ states because of the relative change in potential energy depths; however, these problems are beyond our scope. Here we show a crude model including magnetization tilting and relative portion change to recommend directions for future work. The roughness of domain walls should also be taken into account, but here we ignore this effect.

First, we think about the SOT effect. Supplementary Figure 11 shows an example of domain wall magnetization (solid and dashed pink arrows) and applied H_y. The magnetization angle can have two values ($\varphi + \chi$ and $\varphi - \chi$) at the domain wall because $H_{DMI}^{*}<H_S$. Regarding the $\varphi + \chi$ state (solid pink arrow), an orthogonal component of H_y on the domain wall generates $H_y\sin\psi$ and $H_y\sin\psi$ attempts to rotate the magnetization angle by $(H_y/H_S)\sin\psi$. To prevent this magnetization rotation (or to prevent the SOT field change), the domain wall angle (φ) should be rotated oppositely by $-(H_y/H_S)\sin\psi$. Replacing ψ with $\varphi \pm \chi$ and averaging the wall angle change, we obtain the magnetization tilting effect on the SOT facet equation.

Next, a projection of H_y on the domain wall produces $H_y\cos\psi$, which increases the energy depth of the $\varphi + \chi$ magnetization state. A simple Arrhenius assumption leads to a relative portion of the $\varphi \pm \chi$ state as $e^{(E\varphi\pm\chi/kT)}/\{e^{(E\varphi+\chi/kT)} + e^{(E\varphi-\chi/kT)}\}$, where $E_{\varphi\pm\chi}$ is the energy depth of the $\varphi \pm \chi$ state and kT is the thermal energy. Under the zero in-plane field, $E_{\varphi+\chi}$ and $E_{\varphi-\chi}$ are equal ($=E_0$) and the relative portion is 1/2 for each magnetization state. If the applied in-field strength is small enough, the relative portion goes to $(1+\zeta_{\varphi\pm\chi})/(2+\zeta_{\varphi+\chi}+\zeta_{\varphi-\chi})$ with $E_{\varphi\pm\chi}/kT=E_0/kT+\zeta_{\varphi\pm\chi}$. As a result, the averaged total SOT field is $H_{SOT}(\psi=0)\times[\{\cos(\varphi+\chi)(1+\zeta_{\varphi+\chi})+\cos(\varphi-\chi)(1+\zeta_{\varphi-\chi})\}/\{2+\zeta_{\varphi+\chi}+\zeta_{\varphi-\chi}\}]$, where ζ is proportional to the parallel component of the in-plane field on the domain wall magnetization; however, ζ is a non-dimensional parameter, meaning that an additional factor (r_T) is required to convert the field to ζ. Moreover, r_T should be determined by M_S, wall width, kT, magnetic layer thickness, and an independent segment length of the domain wall. If we obtain a variation of the SOT field as a function of the in-plane magnetic field, we obtain the effect of the relative portion change of the $\varphi \pm \chi$ states on the facet equations.

After some calculations and simplifications, we get the first order facet tilting and sharpening equations for the SOT-only case. Here, we ignore the domain tilting.

$$\frac{\sin \Phi_-}{\cos \Phi_+} \cong \left[\frac{{H_{\mathrm{DMI}}}^*}{{H_\mathrm{S}}^2} + r_\mathrm{T}\left(\frac{H_\mathrm{S}}{{H_{\mathrm{DMI}}}^*} - \frac{{H_{\mathrm{DMI}}}^*}{H_\mathrm{S}}\right)\right] H_x, \tag{21a}$$

$$\frac{\sin(\Phi_+ - \Phi_{+0})}{\sin \Phi_{+0}} \cong \left[\frac{{H_{\mathrm{DMI}}}^*}{{H_\mathrm{S}}^2} + r_\mathrm{T}\left(\frac{H_\mathrm{S}}{{H_{\mathrm{DMI}}}^*} - \frac{{H_{DMI}}^*}{H_\mathrm{S}}\right)\right] H_y. \tag{21b}$$

On the right side of the above equations, the first coefficient of H_x and H_y comes from magnetization tilting and the second term from the change of the relative portion of $\varphi + \chi$ and $\varphi - \chi$ states.

A similar description is possible for the φ=±90° states (Supplementary Note 5). Supplementary Equation (18a) will be changed as follows. Here, we ignore the domain tilting.

$$-\frac{H_{z,\mathrm{offset}}}{H_{\mathrm{SOT},0}} \cong \left[\frac{|{H_{\mathrm{DMI}}}^*|}{{H_\mathrm{S}}^2} + r_\mathrm{T}\left(\frac{H_\mathrm{S}}{|{H_{\mathrm{DMI}}}^*|} - \frac{|{H_{\mathrm{DMI}}}^*|}{H_\mathrm{S}}\right)\right] H_y. \tag{22}$$

This equation explains only the small field range of H_y. The large H_y situation requires a more complicated equation, but we know that $H_{z,\mathrm{offset}}$ will converge to $\sim H_{\mathrm{SOT}}(\psi=0)$ at a large H_y.

We have to point out that these facet equations with a weak DMI have a single coefficient of H_x and H_y, similar to large DMI cases (without domain tilting). Therefore, we can observe facet tilting and sharpening regardless of the DMI strength and use similar interpretations on the experiments by introducing $\widetilde{H}_{\mathrm{DMI}}$ as follows.

$$\widetilde{H}_{\mathrm{DMI}} = \left[\frac{{H_{\mathrm{DMI}}}^*}{{H_\mathrm{S}}^2} + r_\mathrm{T}\left(\frac{H_\mathrm{S}}{{H_{\mathrm{DMI}}}^*} - \frac{{H_{\mathrm{DMI}}}^*}{H_\mathrm{S}}\right)\right]^{-1} \tag{23}$$

For the SMT-only facet, we think about the domain wall type (Bloch or Néel) and their wall width differences. The wall width Δ_w depends on the θ_w (θ_w=0 or 180° for a Bloch wall and θ_w=90 or 270° for a Néel wall), such as $\Delta_\mathrm{w}(\theta_\mathrm{w})=\Delta_\mathrm{B}+\Delta_\mathrm{BN}\cos^2\theta_w$ with a Bloch wall width (Δ_B) and a difference in widths of the Bloch and Néel walls (Δ_BN). At a zero in-plane field, the wall width (Δ_0) is $\Delta_\mathrm{w}(\chi)$. The application of small in-plane fields produces small tilting of θ_w, which results in wall width change. If we average the wall width variation, including the $\psi = \varphi - \chi$ situation, we obtain the following equations for the SMT-only case. In this case, the changes in relative portions of the $\varphi + \chi$ and $\varphi - \chi$ states owing to thermal activation do not produce a first-order contribution.

$$\frac{\sin \Phi_-}{\cos \Phi_+} \cong \frac{\Delta_{\mathrm{BN}} \sin 2\chi \sin \chi}{\Delta_0}\frac{H_x}{H_\mathrm{S}}. \tag{24a}$$

$$\frac{\sin(\Phi_+ - \Phi_{+0})}{\sin \Phi_{+0}} \cong -\left(\frac{1}{\tan^2 \Phi_{+0}}\right)\frac{\Delta_{\mathrm{BN}} \sin 2\chi \sin \chi}{\Delta_0}\frac{H_y}{H_\mathrm{S}}. \tag{24b}$$

This SMT-only facet also has the same forms of equations as the large DMI case if we introduce an effective anisotropy field $\widetilde{H}_K$ as follows.

$$\widetilde{H}_K = \frac{\pi \Delta_0 H_\mathrm{S}}{2\Delta_\mathrm{BN} \sin 2\chi \sin \chi} \tag{25}$$

It should be noted that we know SMT strength depends on wall width, but so does β; thus, $\widetilde{H}_K$ should be replaced with $\widetilde{H}_K{}^*$.

It is well known that the Sub/Ta(5 nm)/Pt(2.5 nm)/Co(0.35 nm)/Pt(1.5 nm) structure[16] exhibits a small DMI, a non-zero SOT, and a dominant SMT, so we test a sample 3 mm in width and 0.6 mm in length. We obtain the $H_\mathrm{DMI}{}^*$ (96±10 Oe) by asymmetric expansion of the circular domain (Supplementary Fig. 12a) at a sample temperature of ~325 K using a heater. The sign of $H_\mathrm{DMI}{}^*$ is opposite the Pt/CoFeB/MgO structures shown in the above sections. We also obtain H_K (13±1 kOe) at the same temperature. Then, we turn off the heater and turn on the constant current with 0.35 A that maintains the sample temperature. Before the facet experiments, we obtain H_ST as a function of H_y under ±0.35 A by employing the DW motion measurements typically used in the wire structure[16] because the film structure also makes it possible to observe the DW motions. We nucleate circular domains by pulsed H_z under fixed H_y and I. Then, we tune the offset field of H_z for stopping the DW motions at each part of the domain wall with φ=0° and φ=180°. We add a sinusoidal oscillation to H_z to amplify the wall motions. Supplementary Figure 12 is the result of averaged H_ST from the +0.35 A and –0.35 A. H_ST shows a similar tendency, as already shown in Ref. 16. From the results, we obtain $H_\mathrm{ST,0}$=–2.8±0.1 Oe, $H_\mathrm{SOT,0}$=+0.7±0.2 Oe, $H_\mathrm{SOT}(\psi=0)$=–5.3±0.3 Oe, $H_\mathrm{SMT,0}$=–3.6±0.2 Oe, and H_S=0.66±0.05 kOe.

Next, we perform facet experiments with the same current. The linear relation between $\cos\Phi_+$ and H_z means that $H_\mathrm{ST,0}$=–2.7±0.1 Oe, which well fits the wall motion experiments (Supplementary Fig. 12b). In Supplementary Fig. 12c, $H_{z,\mathrm{offset}}$ has a saturation value (=–5.4±0.2 Oe) almost identical to the $H_\mathrm{SOT}(\psi=0)$ obtained by the DW motion measurements. Near H_y=0, $H_{z,\mathrm{offset}}$ does not show a sudden jump, which means that there are several effects, such as wall magnetization tilting, thermal activation, and domain wall roughness, as we expected. Employing our crude model, we get $H_\mathrm{SOT,0}/\widetilde{H}_\mathrm{DMI}$=–0.007±0.0003. Supplementary Figure 12d and e shows the tilting data with no meaningful angle variations, but sharpening shows a clear angle variation. Therefore, we expect that the SOT on the facet tilting is almost compensated by the SMT effect ($H_\mathrm{SOT,0}/\widetilde{H}_\mathrm{DMI} + H_\mathrm{SMT,0}/$

$\widetilde{H}_K{}^* \approx 0$). If we use obtained values from wall motion experiments, we get $\widetilde{H}_{\mathrm{DMI}}$~100 Oe, $\widetilde{H}_K{}^*$~510 Oe, and r_{T}~0.0014.

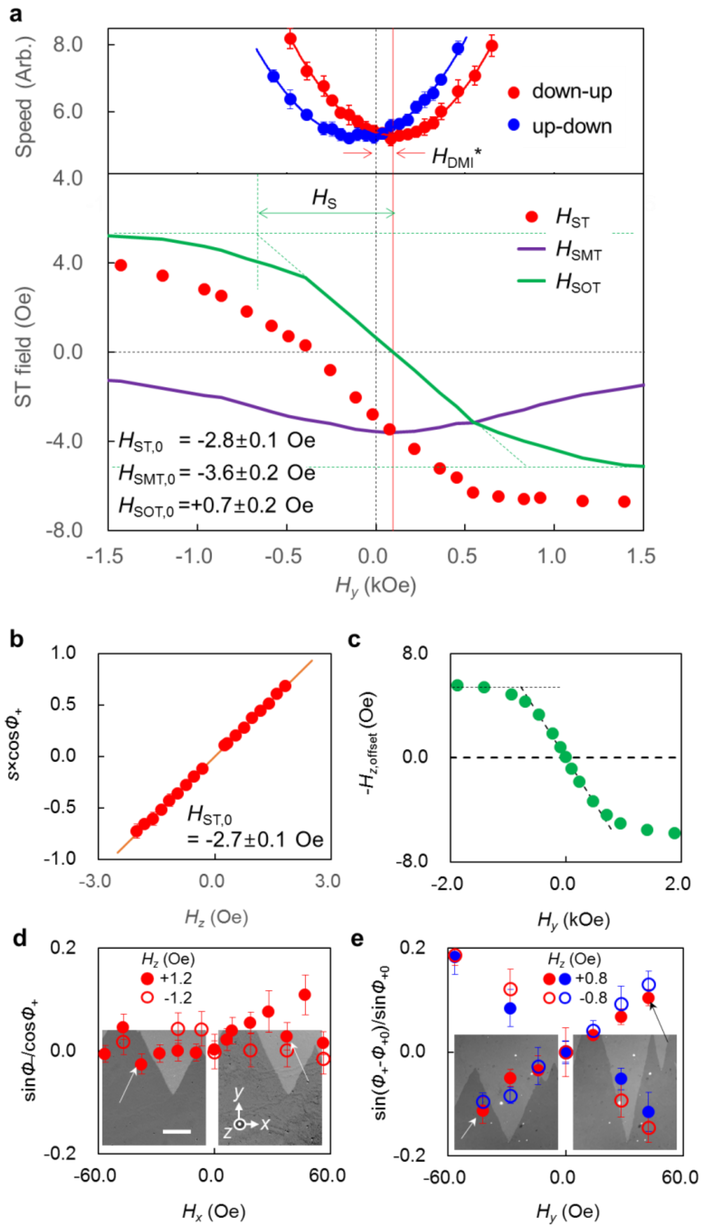

Supplementary Figure 12 | Experiments on Sub/Ta(5 nm)Pt(2.5 nm)/Co(0.35 nm)/Pt(1.5) stack. a, Asymmetric expansion speeds of the circular domain and spin-torque field measurements. **b,** Facet experiment for detecting $H_{\mathrm{ST},0}$. **c,** $H_{z,\mathrm{offset}}$ as a function of H_y. **d,** Tilting experiments with I=+0.35 A. **e,** Sharpening results. I=+0.35 A for red symbols and I=–0.35 A for blue symbols. Inset images show facet domains with different field conditions as indicated by arrows. The scale bar is 100 μm. Error bars are the standard deviation of five measurements.

Supplementary References

1. Li, Z. & Zhang, S. Domain-wall dynamics driven by adiabatic spin-transfer torques. *Phys. Rev. B* **70,** 024417 (2004).
2. Thiaville, A., Nakatani, Y., Miltat, J. & Suzuki, Y. Micromagnetic understanding of current-driven domain wall motion in patterned nanowires. *Europhys. Lett.* **69,** 990–996 (2005).
3. Ryu, J., Choe, S.-B., & Lee, H.-W. Magnetic domain-wall motion in a nanowire: depinning and creep. *Phys. Rev. B* **84,** 075469 (2011).
4. Burrowes, C. *et al*. Non-adiabatic spin-torques in narrow magnetic domain walls. *Nat. Physics* **6,** 17–21 (2010).
5. Khvalkovskiy, A. V. *et al*. Matching domain-wall configuration and spin-orbit torques for efficient domain-wall motion. *Phys Rev B* **87,** 020402 (2013).
6. Yu, J. *et al.* Spin orbit torques and Dzyaloshinskii-Moriya interaction in dual-interfaced Co-Ni multilayers. *Sci. Rep.* **6,** 32629 (2016).
7. Liu, L., Lee, O. J., Gudmundsen, T. J., Ralph, D. C. & Buhrman, R. A. Current-induced switching of perpendicularly magnetized magnetic layers using spin torque from the spin Hall effect. *Phys. Rev. Lett*. **109,** 096602 (2012).
8. Boulle, O. *et al*. Domain wall tilting in the presence of the Dzyaloshinskii-Moriya interaction in out-of-plane magnetized magnetic nanotracks. *Phys. Rev. Lett*. **111,** 217203 (2013).
9. Ryu, K.-S., Thomas L., Yang, S.-H. & Parkin S. Chiral spin torque at magnetic domain walls. *Nat. Nanotech*. **8,** 527–533 (2013).
10. Miron, I.-M. *et al*. Fast current-induced domain-wall motion controlled by the Rashba effect. *Nat. Mater.* **10,** 419–423 (2011).
11. Haazen, P. P. J. *et al.* Domain wall depinning governed by the spin Hall effect. *Nat. Mater.* **12,** 299–303 (2013).
12. Je, S.-G. *et al*. Asymmetric magnetic domain-wall motion by the Dzyaloshinskii-Moriya interaction. *Phys. Rev. B* **88,** 214401 (2013).
13. Dzyaloshinskii, I. E. Thermodynamic theory of weak ferromagnetism in antiferromagnetic substances. *Sov. Phys. JETP* **5,** 1259–1272 (1957).
14. Moriya, T. Anisotropic superexchange interaction and weak ferromagnetism. *Phys. Rev.* **120,** 91–98 (1960).

15. Jué, E. *et al.* Domain wall dynamics in ultrathin Pt/Co/AlOx microstrips under large combined magnetic fields. *Phys. Rev. B* **93,** 014403 (2016).

16. Je, S.-G. *et al.* Emergence of huge negative spin-transfer torque in atomically thin Co layers. *Phys. Rev. Lett*. **118,** 167205 (2017).

17. Bohlens, S. & Pfannkuche, D. Width dependence of the nonadiabatic spin-transfer torque in narrow domain walls. *Phys. Rev. Lett.* **105,** 177201 (2010).

18. Vanhaverbeke, A. & Viret, M. Simple model of current-induced spin torque in domain walls. *Phys. Rev. B* **75,** 024411 (2007).

19. Jué, E. *et al.* Chiral damping of magnetic domain walls *Nat. Mater.* **15,** 272-277 (2016).
